# Integrating technology and environmental data to predict mismanaged plastic waste in a watershed

**DOI:** 10.1111/jiec.70093

**Published:** 2025-09-04

**Authors:** Lara M. Pinheiro, Diana Ita-Nagy, Desireé G. Hidalgo, Daniela Flor, Andrea Osorio Baquero, Nicole Becerra, Inty Grønneberg, Ian Vázquez-Rowe, Ramzy Kahhat, Ceri Lewis, Tamara S. Galloway

**Affiliations:** 1 Faculty of Health and Life Sciences, University of Exeter, Stocker Road, EX4 4QD Exeter, UK; 2 Peruvian Life Cycle Assessment & Industrial Ecology Network (PELCAN), Department of Engineering, Pontificia Universidad Católica del Perú (PUCP), San Miguel, Lima, Peru; 3 Ichthion Limited, Staines Upon Thames, UK

**Keywords:** industrial ecology, macroplastics, material flow analysis, plastic transport, river clean-up, waste management

## Abstract

**Comprehensive** methods for estimating mismanaged waste accumulation in the environment are limited, especially in the Global South, and new technologies are urgently needed. Here, we applied the Azure system, a physical floating barrier designed to retain and extract river floating waste while providing observational data of mismanaged waste, comparing results with a modeling tool that uses material flow analysis to provide estimates of mismanaged waste, incorporating environmental and socioeconomic factors. The Azure system was installed at the Portoviejo River (Ecuador), and anthropogenic litter was removed, extracted, weighed, and classified. Approximately 13.8 tonnes (t) of litter were collected over 2 years of sampling, of which 87% were plastic bags containing domestic waste. About 45% of the total waste collected, that is, 6.2 t, was estimated to be plastic waste. In contrast, modeled mismanaged plastic waste estimates for the Portoviejo River varied between 148 and 1858 t per year, at least two orders of magnitude higher than field data. These results highlight the discrepancy that can occur between observational data and waste estimates. The factors that contribute to this are discussed here to help understand riverine waste sources and transport to the ocean. The results emphasize the need for a better understanding of socioeconomic and environmental aspects in the Global South to help the development of better modeling tools. Our findings of domestic deposition as a major source of riverine contamination in the Portoviejo watershed emphasize the importance of waste management for tackling river contamination. Effective monitoring tools, such as the Azure system, could help improve this.

**Supplementary Information:**

The online version of this article (doi:10.1111/jiec.70093) contains supplementary material, which is available to authorized users.

## INTRODUCTION

The generation of anthropogenic solid waste is an intrinsic consequence of human activities and is on the rise (Lebreton & Andrady, 2019). Global waste generation was around 20 billion tonnes (t), or 2.6 t per capita per year in 2017, and is expected to increase to 46 billion t by 2050 if no mitigation measure is taken (Maalouf & Mavropoulos, 2023). Such quantities put waste management systems in different countries under pressure, especially considering that waste collection can be heterogeneous even inside the same country. One study showed great differences between developed, developing, and less developed countries in terms of sources, governance, public awareness, and technologies and infrastructure available for waste management (Mmereki et al., 2016). Consequently, it is estimated that the world generated over 500,000 t of mismanaged waste in 2015 (Lebreton & Andrady, 2019). The positive correlation between urbanization in developing nations and the generation of mismanaged waste can be explained by their rapid but disorganized growth. In the case of Latin America and the Caribbean, 40 million people still lack access to proper waste collection (UN Environment, 2018), resulting in 50% of all municipal solid waste (MSW) being mismanaged (Margallo et al., 2019), released, and accumulated in nature. Considering that up to 12% of a country's MSW can be composed of plastic waste (UNEP et al., 2018), which in turn might not undergo proper disposal, plastics have become a concerning topic in environmental pollution as large quantities have been reported in aquatic systems such as rivers (van Emmerik & Schwarz, 2020), lakes and reservoirs (Nava et al., 2023), estuaries (Pinheiro et al., 2021), and finally the ocean (Eriksen et al., 2023). Plastic materials have the potential to reach and cause deleterious effects to the environment (Bhat et al., 2022; Siddiqua et al., 2022), so addressing plastic pollution has become a major priority, as documented in the UN Ocean Decade Challenges (See Hatje et al., 2024) and the UN Sustainable Development Goals (https://sdgs.un.org/goals, SDG 12 and 14).

There is a need to understand and estimate the amount of waste transported and accumulated in the different natural compartments (land, freshwater, and ocean), especially plastics, as it can be transported from land-based sources via rivers to estuaries and then to the ocean (Lima et al., 2020). Riverine plastic transport is the main contributor to ocean plastic emissions (Lebreton & Andrady, 2019), even though estimations do not equally portray scenarios from different watersheds in the world. Estimations based on observational data and/or modeling show plastic riverine transport to oceans to be between 0.41 and 4 × 10^6^ t year^−1^ (Schmidt et al., 2017), 57 and 265 million t year^−1^ (Mai et al., 2020), 0.8 and 2.7 million t year^−1^ (Meijer et al., 2021), or 0.5 million t year^−1^ (Strokal et al., 2023). These evaluations use socioeconomic parameters such as estimates of plastic waste generation, waste management capacities, gross domestic product (GDP), and population (Lebreton & Andrady, 2019), while some also consider environmental parameters such as river hydrology (Lebreton et al., 2017) and meteorological regimes (Meijer et al., 2021). Parameters such as accumulation zones caused by natural and artificial barriers that favor solid waste retention inside or around rivers, that is, in their watershed, remain understudied even though current evidence supports the retention of solid waste, namely plastics, in coastal watersheds (Ita-Nagy et al., 2022; Strokal et al., 2023; van Emmerik et al., 2022). Ita-Nagy et al. (2022) proposed a new method for estimating regional riverine plastic emissions, which includes natural and anthropogenic physical barriers and the removal of waste through recycling, to account for observational data gaps, including overestimation of plastic exports to the ocean.

The available studies modeling world riverine input of plastic to the oceans do not currently include empirical data from South American (SA) rivers, even though they are responsible for approximately 30% of global freshwater discharge to oceans, that is, around 10,800 km^3^ year^−1^ (Milliman & Farnsworth, 2011). The estimations of riverine export of plastic contamination made by Meijer et al. (2021) added observational data from Asian rivers to existing data for European and North American rivers cited by Lebreton et al. (2017) and Schmidt et al. (2017). Even though South American rivers are included in estimations based on socioeconomic data, observational data on plastic contamination were not considered. Similarly, the work by Strokal et al. (2023) applied the MARINA-Multi model for world riverine plastic export, but again lacked observational data for SA. Another example of a gap relates to methodologies. A recent review on methods for measuring macroplastics (>25 mm) in rivers by Hurley et al. (2023) used a relatively broad literature search method, but they did not include any work in SA. This might be because many studies in SA focus on coastal regions or on microplastics (<5 mm) only (see Orona-Návar et al., 2022), which was an exclusion criterion for Hurley et al. (2023). Similarly, Morales-Caselles et al. (2021) use no data on SA rivers in their global analysis of aquatic litter. This highlights the originality of the present work that feeds into future studies with essential empirical data in an understudied geographical area.

A range of technologies for plastic remediation has been developed around the world and placed in aquatic systems, attempting to remove anthropogenic litter from the environment. This involves both prevention and cleanup technologies, which target different aquatic environments and litter types (Falk-Andersson et al., 2023). Among cleanup devices, there are barrier technologies such as The Great Bubble Barrier (https://thegreatbubblebarrier.com/), which uses a bubble curtain to direct litter to a catchment system. Other barriers use a containment barrier to either trap or direct floating materials to a collection point, for example: the intermittent barriers from Mr. Trash Wheel^®^ (https://www.mrtrashwheel.com/), the CLEAN (CLAIM's Litter Entrapping Autonomous Network) TRASH (Tactical Recover Accumulation System Hellas) system (https://www.oilspillresponse.gr/service-item.php?sid=1), and a family of barrier technologies from The Ocean Cleanup initiative (https://theoceancleanup.com/). There is a great potential to use these technologies for long-term monitoring of riverine waste flow, considering the inherent difficulty in collecting robust, consistent data on riverine waste export from large-sized river watersheds.

This study aims to generate empirical data from the deployment of a local-scale cleanup device to assess the amount of mismanaged plastic waste flowing towards the Pacific Ocean through the Portoviejo River watershed in Ecuador. Empirical data of riverine litter were obtained with a cleanup technology called the Azure system, developed by Ichthion Limited (https://ichthion.com/technology/), a startup company working on solutions to reduce plastic waste in the Eastern Pacific through technology and community action. Results were additionally compared to modeled data estimating plastic waste flow obtained using the novel methodology described by Ita-Nagy et al. (2022). Ultimately, the case study reports on how to monitor, quantify, and characterize solid waste contamination in a South American river, comparing observational data with modeled estimates.

## METHODS

### Study area: The Portoviejo River watershed

**FIGURE 1:**
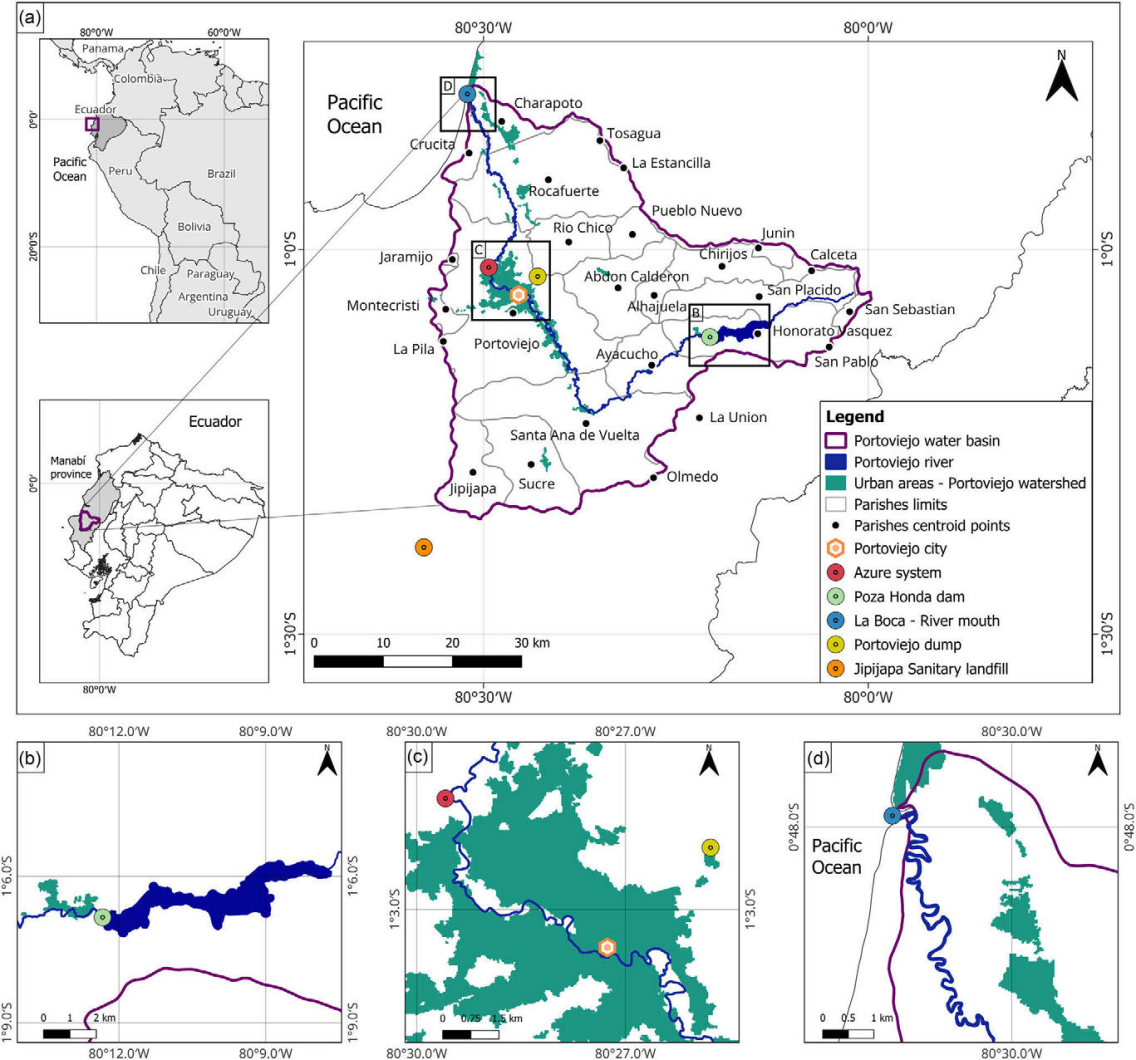
(a) Detailed map of the Portoviejo water basin in Ecuador showing the location of the Azure System in the Portoviejo River at the city of Portoviejo (Ecuador) and the parishes (*parroquias*) composing the watershed, and close-ups of the water basin at: (b) the Poza Honda dam, (c) the Portoviejo urban area, and (d) the river mouth.

The Portoviejo River was chosen due to its medium size as a pilot installation of the Azure system (Figure 1a). It runs along the coastal region of Ecuador, in South America, in the province of Manabí, with a length of 130.79 km from Poza Honda dam (Figure 1b), located 30 km from the city of Portoviejo. The river passes through the city of Portoviejo (Figure 1c) until the river mouth at La Boca (Figure 1d). The Portoviejo water basin covers an area of approximately 2080 km^2^ and has irregular topography in most of its territory, which favors soil erosion along the water courses (Cuenca Zambrano & Pacheco Gil, 2021).

Rainfall patterns follow a marked dichotomy between rainy and dry seasons. The dry season normally occurs between July and November, while the rainy season occurs between December and June (Mendoza Alava et al., 2022). Flooding events are common in Manabí during the rainy season (IFRC, 2023), which are frequently associated with the annual latitudinal fluctuation of the Intertropical Convergence Zone (ICZ) and the El Niño Southern Oscillation (ENSO) (Guerrero et al., 2022). These regions are prone to flooding due to the presence of large plains along river courses, which represents a risk for human settlements concentrated in these areas (Sandoval Erazo et al., 2022) (see more in M1 of Supporting information S1).

### Empirical data collection: The Azure system

**FIGURE 2:**
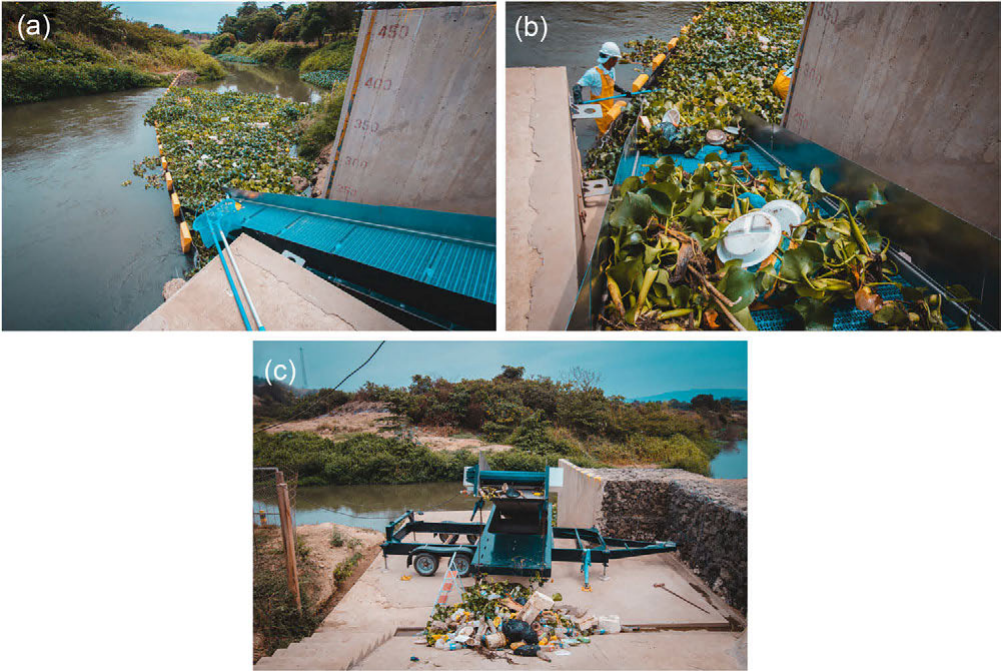
The Azure barrier system installed at the Portoviejo River, Ecuador. (a) The operating barrier; (b) operators directing litter to the conveyor belt for removal; (c) litter removed from the Portoviejo River. *Source*: Photos: Andrea Osorio Baquero.

The Azure system was implemented in 2020 by Ichthion Limited as an extraction tool for anthropogenic waste in river systems. It consists of a physical, floating barrier that collects large, suspended materials that are being transported down the river (Figure 2a). The system is designed to extract up to 80 t per day of floating litter from the river course, which so far has never been reached. In addition to a floating barrier that traps materials going down the river surface up to 60 cm depth (i.e., 18%–75% of river depth on installation point), the system also has a conveyor belt that removes the trapped material into a sorting area onshore.

The Azure system has been operative in the Portoviejo River at 1° 01′ 23.9″ S, 80° 29′ 35.6″ W, downstream from the city of Portoviejo (Figure 1c). Litter collection is led by field engineer officers and operators from Ichthion and usually happens daily on weekdays. For operator and machinery safety reasons, the Azure system was dismounted during days of strong river flow and height (over 4.5 m) around March/April, and therefore, litter was not collected on those days. This resulted in an average of 35% fewer operative days in these 2 months than in the others over 2 years of analysis. Figure 2 illustrates the operation of the Azure system. For litter removal, the conveyor belt is switched on and operators enter the river to manually direct litter to the ramp (Figure 2b), depending on the river level. Once the litter is out of the water (Figure 2c), all the vegetation trapped is separated from the anthropogenic litter and returned to the river after the conveyor belt. Anthropogenic litter was weighed (dry weight) using a portable weighing scale and classified according to categories adapted from the OSPAR Guidelines Edition 1.0 (OSPAR Commission, 2010). Data collection for litter weight and categories happened once a week from January 2021 until December 2022, for a total of 105 data points. The content of filled plastic bags was not analyzed due to sanitary risks involved, but the amount of plastic inside those bags was calculated as 8.7%–9.0% of its total weight, as per estimates of plastic content of a typical household solid waste bag in Ecuador and other countries in Latin America and the Caribbean (Diéguez-Santana et al., 2021; Hidalgo et al., 2019) which is shown not to vary considering different data sources and temporal variability (Margallo et al., 2019). Upon data collection, all recyclable litter was destined to be recycled or repurposed to benefit the local community through the project *Fundación Circular* (https://somoscircular.org/proyectos/ecowork-ii/).

### Meteorological and hydrological data

To investigate any relationships between litter quantities and environmental factors, data on meteorological data (precipitation [mm day^−1^], average temperature [°C], wind speed [m.s^−1^], and wind direction [cardinal directions]) and hydrological data (river flow velocity [m s^−1^], river discharge [m^3^ s^−1^], river depth [cm], and river width [m]) were collected (see M2 of Supporting information S1).

### Litter empirical data analysis

Litter data from February 2021 until December 2022 was reported as average mass (kg) per operative day by taking the average mass (kg) collected per week for each month, divided by the average operative days per week for each month. Standard deviation values were indicated when citing averages. The quantities of litter for each litter category were reported as a percentage of occurrences for each month. Statistical analyses were performed in the PAST software (version 4.10) (Hammer et al., 2001) and are fully described in M3 of Supporting information S1.

### Model for estimating plastic waste toward the ocean (pWtO)

To model the quantities of plastic waste being transported toward the ocean (pWtO), the methodology developed by Ita-Nagy et al. (2022) (Equation 1) was used as a comprehensive approach considering a range of factors in the estimation of waste transport. Prior to selecting the factors, we defined the study area for equation input as the hydrological region of the Portoviejo River basin, as per Mendoza Alava et al. (2022), that is, level 4 hydrological units (SENAGUA, 2011). This includes all municipalities within the Portoviejo River catchment, from its origin at the Poza Honda Dam until its estuarine region at La Boca (Figure 1).

Equation (1) shows the calculation of plastic waste toward the ocean (pWtO) generated from municipal solid waste (MSW) in a watershed proposed by Ita-Nagy et al. (2022).

1 $${\mathrm{pWtO}} = \sum{Q_{{\mathrm{mMSW}}{p_{{\mathrm{ij}}}}}}*\left( {1 - {f_{{\mathrm{s}}{{\mathrm{r}}_i}}}} \right)*\left\{ { \def\eqcellsep{\;}\begin{array}{@{}*{1}{c}@{}} {{f_{{\mathrm{c}}{{\mathrm{l}}_i}}},}\\ {{f_{{\mathrm{c}}{{\mathrm{i}}_i}}},}\\ {{f_{{\mathrm{c}}{{\mathrm{w}}_i}}}*{f_{{\mathrm{r}}{{\mathrm{s}}_j}}}*\left( {{{\Pi}}_{{\mathrm{ca}} = 0}^z{f_{{\mathrm{ca}}}}} \right),} \end{array} } \right. \def\eqcellsep{\;}\begin{array}{@{}*{2}{c}@{}} {}\;{{\mathrm{if\;}}0 < \bar x < 0.1\;{\mathrm{km}}}\\ {}\;{{\mathrm{if\;}}\bar x > 0.1\;{\mathrm{km\;in\;an\;inter}} - {\mathrm{basin}}}\\ {}\;{{\mathrm{otherwise}}} \end{array}$$

The first parameter in the equation depicts the total amount of mismanaged plastic from MSW of a specific urban agglomeration *i* placed in a river basin *j* (QmMSWp). This was calculated using the latest available information on population census (2022) and on waste management from the Solid Waste Management (*Gestión de Resíduos Sólidos*, GRS), both provided by the National Institute of Statistics and Censuses (*Instituto Nacional de Estadística y Censos*, INEC). When available, data were used at the parish level (*parroquia* in Spanish), the third-level administrative units in Ecuador. For waste production per capita, waste management, and recycling rates, data were only available at the province level (*cantón*, first-level administrative unit in Ecuador) (see M4 of Supporting information S1).

Each factor *f* in the equation expresses the effects of waste recovery (*f*_sr_), the distance (*x*) to the coastline (*f*_cl_), the main river in the watershed (*f*_cw_), or its location in an inter-basin (*f*_ci_). As the equation was applied to the inner area of the watershed only (up until the Azure system point, not the coast), *f*_cl_ and *f*_ci_ were not accounted for. The model also accounts for the effects of river seasonality (*f*_rs_) and different barriers located along the transportation path toward the ocean (*f*_ca_). Natural and man-made infrastructures are analyzed as barriers or boosters of waste mobilization, reducing, or impeding its transportation toward the ocean. Thus, the model becomes more holistic and site-specific. The Azure barrier was accounted for as a man-made barrier in the calculations. Full description of the factors can be found in Table M1 of Supporting Information S1, adapted from Ita-Nagy et al. (2022).

In this study, the term “retention” refers to anthropogenic waste retained inland, that is, not reaching the river course. This is important to define as three scenarios were considered for estimating QmMSWp for each municipality, representing different rates of inland waste retention according to different waste dissipation coefficients attributed to natural and anthropogenic barriers by Ita-Nagy et al. (2022). The upper scenario uses conservative retention capacities per identified barrier, while the lower scenario estimates a higher retention rate. The coefficients also allow the user to analyze the effect of barriers such as the Azure cleanup device on waste mobilization (Table M1 of Supporting Information S1 and Table M2 of Supporting Information S2).

## RESULTS

### Riverine litter, hydrological, and meteorological data

**FIGURE 3:**
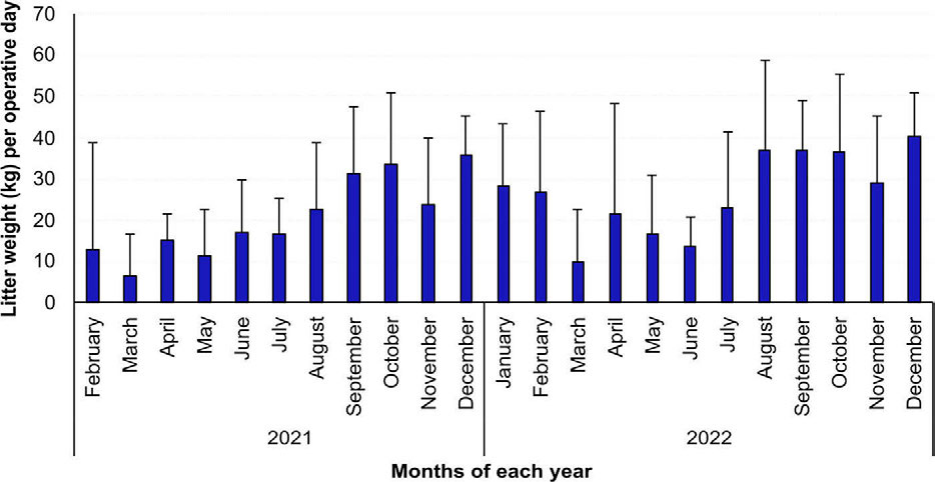
Measured average weight (average kg + standard deviation) per operative day collected by the Azure system from February 2021 to December 2022. Underlying data for this figure are available in the data repository at https://doi.org/10.5285/e78e1cef-e30b-4313-8733-c03e1a7b7b2f.

Over 13 tonnes (t) of litter were collected by the Azure system from the Portoviejo River in 2021 and 2022. The average litter quantities collected were 23.8 ± 10.1 kg day^−1^. The month with maximum litter collection was December 2022 with 40.2 ± 10.5 kg day^−1^, while the minimum litter collection was performed in May 2021 with 11.4 ± 11.3 kg day^−1^ (Figure 3). The average quantity of litter collected by the Azure system in 2021 and 2022 was 603.2 ± 318.4 kg per month, with a total of 5.7 t in 2021 and 8.1 t in 2022. The two-way ANOVA test detected significant differences between months (*F*_10,88 _= 3.027, *p* = 0.002) but not between years (*F*_1,88_ = 3.019, *p* = 0.086; Table S1 of Supporting Information S1), but the Tukey's test revealed that the latter was restricted to differences between March and October/December. No significant differences in average litter quantities per month were found when analyzing the interaction between sampling months and sampling years (*F*_10,88_ = 0.197, *p* = 0.996), but a significant difference (*F*_1,107.9 _= 5.954; *p* = 0.016) was found between rainy (December–June) and dry seasons (July–November) even without considering March and April (*F*_1,72.47 _= 7.976, *p* = 0.006) when the system was not operative for the whole month due to heavy rainfall. Average precipitation varied from 2.1 ± 2.1 mm day^−1^ in the rainy season (December–June) to 0.01 ± 0.02 mm day^−1^ in the dry season (July–November), with the highest average in March for both years (Table 1). River parameters of flow velocity, discharge, depth, and width had higher average values in the rainy season when compared to the dry season (Table 1).

**TABLE 1:** Hydrological (avg ± stdev) and meteorological (avg) data collected at the Azure system collection point and provided by the Ecuadorian National Institute of Meteorology and Hydrology (*Instituto Nacional de Meteorología e Hidrología*—INAMHI), respectively.

		**Collected data**					**INAHMI data**		
**Year**	**Month**	**Average river flow velocity (m s** ^**-1**^ **)**	**Average river discharge (m** ^**3**^ ** s** ^**-1**^ **)**	**Average precipitation (mm day** ^**-1**^ **)**	**Avg of river depth (cm)**	**Avg of river width (m)**	**Average temperature (°C)**	**Average wind speed (m s** ^**-1**^ **)**	**Average wind direction**
2021	Jan	0.65 ± 0.18	18.08 ± 7.65	4.99 ± 5.24	195.28 ± 25.79	22.91 ± 3.10	25.9	1.8	N
	Feb	0.98 ± 0.14	32.69 ± 16.48	2.84 ± 4.16	216.96 ± 49.33	24.33 ± 4.11	26.5	1.3	N
	Mar	0.94 ± 0.15	46.23 ± 38.23	6.34 ± 6.92	319.52 ± 98.39	31.13 ± 8.86	26.2	1.8	S
	Apr	0.89 ± 0.20	37.40 ± 26.75	2.25 ± 2.00	223.48 ± 68.41	24.32 ± 4.56	26.5	1.3	N
	May	1.00 ± 0.14	25.90 ± 10.18	0.64 ± 1.17	185.48 ± 32.35	21.64 ± 3.42	25.6	1.5	N
	Jun	0.74 ± 0.07	12.13 ± 2.07	0.22 ± 0.70	141.84 ± 8.20	16.92 ± 0.82	25.1	1.5	E
	Jul	0.62 ± 0.07	9.10 ± 1.04	0.00 ± 0.00	124.88 ± 5.76	15.37 ± 0.54	24.8	2	E
	Aug	0.50 ± 0.03	6.72 ± 0.98	0.00 ± 0.00	117.62 ± 4.51	14.71 ± 0.41	24.5	1.6	E
	Sep	0.50 ± 0.05	6.15 ± 1.43	0.00 ± 0.00	112.50 ± 5.00	14.25 ± 0.46	25.2	1.1	E
	Oct	0.51 ± 0.04	6.29 ± 1.11	0.00 ± 0.00	114.50 ± 7.68	14.43 ± 0.73	25.2	1.5	E
	Nov	0.57 ± 0.02	7.94 ± 0.56	0.03 ± 0.12	127.50 ± 2.57	15.63 ± 0.24	24.6	1.3	N
	Dec	0.58 ± 0.03	8.12 ± 0.78	0.02 ± 0.11	128.81 ± 4.65	15.73 ± 0.43	25.2	1.3	N
2022	Jan	0.58 ± 0.04	8.59 ± 1.08	1.33 ± 1.85	131.38 ± 10.08	15.98 ± 0.96	25.9	1.8	N
	Feb	0.59 ± 0.04	9.28 ± 2.30	1.55 ± 1.51	136.32 ± 17.19	16.49 ± 1.88	25.6	1.6	E
	Mar	0.90 ± 0.19	46.13 ± 41.91	5.35 ± 3.51	263.55 ± 109.76	27.37 ± 9.01	26.5	1.6	E
	Apr	0.85 ± 0.13	N/A	2.78 ± 4.62	212.00 ± 77.45	N/A	26.4	1.6	E
	May	0.74 ± 0.06	N/A	1.06 ± 1.80	152.08 ± 17.89	N/A	25.5	1.7	E
	Jun	0.71 ± 0.03	N/A	0.00 ± 0.00	140.12 ± 9.50	N/A	24.4	1.2	N
	Jul	0.71 ± 0.03	N/A	0.00 ± 0.00	140.12 ± 9.50	N/A	24.3	1.2	E
	Aug	0.64 ± 0.04	N/A	0.06 ± 0.21	134.66 ± 8.50	N/A	24.7	1.4	E
	Sep	0.57 ± 0.03	N/A	0.00 ± 0.00	85.48 ± 55.61	N/A	24.6	1.6	E
	Oct	0.50 ± 0.03	N/A	0.00 ± 0.00	79.19 ± 55.59	N/A	24.6	1.8	N
	Nov	0.49 ± 0.02	N/A	0.03 ± 0.13	118.50 ± 5.70	N/A	24.7	1.9	E
	Dec	0.47 ± 0.02	N/A	0.08 ± 0.24	120.98 ± 4.38	N/A	25.8	2.1	N

*Note*: N/A indicates months where data were not collected.

As shown in Table 2, most of the mass of material collected (>87%) corresponded to filled plastic bags used for discarding domestic waste. Considering that around 9% of household waste content in Ecuador is plastic (Diéguez-Santana et al., 2021; Hidalgo et al., 2019), it was calculated that 1.09 t of plastic waste was recovered from filled shopping bags over 2 years. Adding that to the total weight of plastic items collected according to the OSPAR categories used in this study (around 5.1 t), we estimate around 6.2 t of plastic waste removed by the Azure system from the Portoviejo River in 2021 and 2022, 44.7% of all material types. Other common types of waste collected were glass bottles (2%), plastic drinks (1.6%), organic waste (1.2%), and clothing (1%) (Table 2). The other 27 categories represented each less than 1% of the total mass collected. A SIMPER analysis of contributions related to weight (kg) showed that filled shopping bags contributed the most to the dissimilarity between months (77.5%), followed by organic waste (4.1%), plastic drink containers (2.9%), glass bottles (2.5%), and empty shopping bags (2.2%) (Table S2 of Supporting Information S1). The two-way ANOSIM test revealed that differences were significant between months but not years (*p*_month _< 0.001 and *p*_year _= 0.554). However, a PERMANOVA test following a PCA (PC1: 99.25% variance explanation) showed significant differences between both sampling years and months alone but not their interaction (Figure 1 and Table S3 of Supporting Information S1). The test showed that differences in litter categories are mostly between March—July and August—January (Table 3 of Supporting Information S1).

**TABLE 2:** Contribution of each category of anthropogenic litter collected by the Azure system in 2021 and 2022 at the Portoviejo River.

OSPAR litter categories	Weight of litter collected (kg)	Contribution to total litter collected (%)
Bags (shopping)—filled	12182.391	87.807
Organic waste	286.01	2.061
Bottles—glass	228.945	1.650
Drinks (bottles, containers, and drums)	179.44	1.293
Plastic/polystyrene pieces	147.734	1.065
Clothing	140.245	1.011
Food containers including fast food containers	133.41	0.962
Foam sponge	108.095	0.779
Other bottles, containers, and drums	73.875	0.532
Cartons, e.g., Tetra Pak (other)	72.76	0.524
Bags (shopping)—empty	59.06	0.426
Jute sack—filled	55.024	0.397
Cosmetics (bottles and containers, e.g., sun lotion, shampoo, shower gel, and deodorant)	42.86	0.309
Other textiles	37.03	0.267
Packaging, plastic sheeting	28.77	0.207
Food cans	21.4	0.154
Other sanitary items	21.1	0.152
Other metal pieces	8.39	0.060
Plastic sheeting	6.71	0.048
Other	6.5668	0.047
Cutlery/trays/straws	4.95	0.036
Toys and party poppers	4.7	0.034
Hard hats	4.6	0.033
Drink cans	4.124	0.030
Face mask	3.72	0.027
Light bulbs/tubes	3.67	0.026
Paraffin or wax pieces	3.2288	0.023
Cardboard	2.2	0.016
Aerosol/spray cans	2.1	0.015
Cups	0.51	0.004
Caps/lids	0.45	0.003

*Note*: Litter categories are adapted from the OSPAR Guidelines Edition 1.0 (OSPAR Commission, 2010).

### Modeled mismanaged waste and plastic waste toward the ocean

**FIGURE 4:**
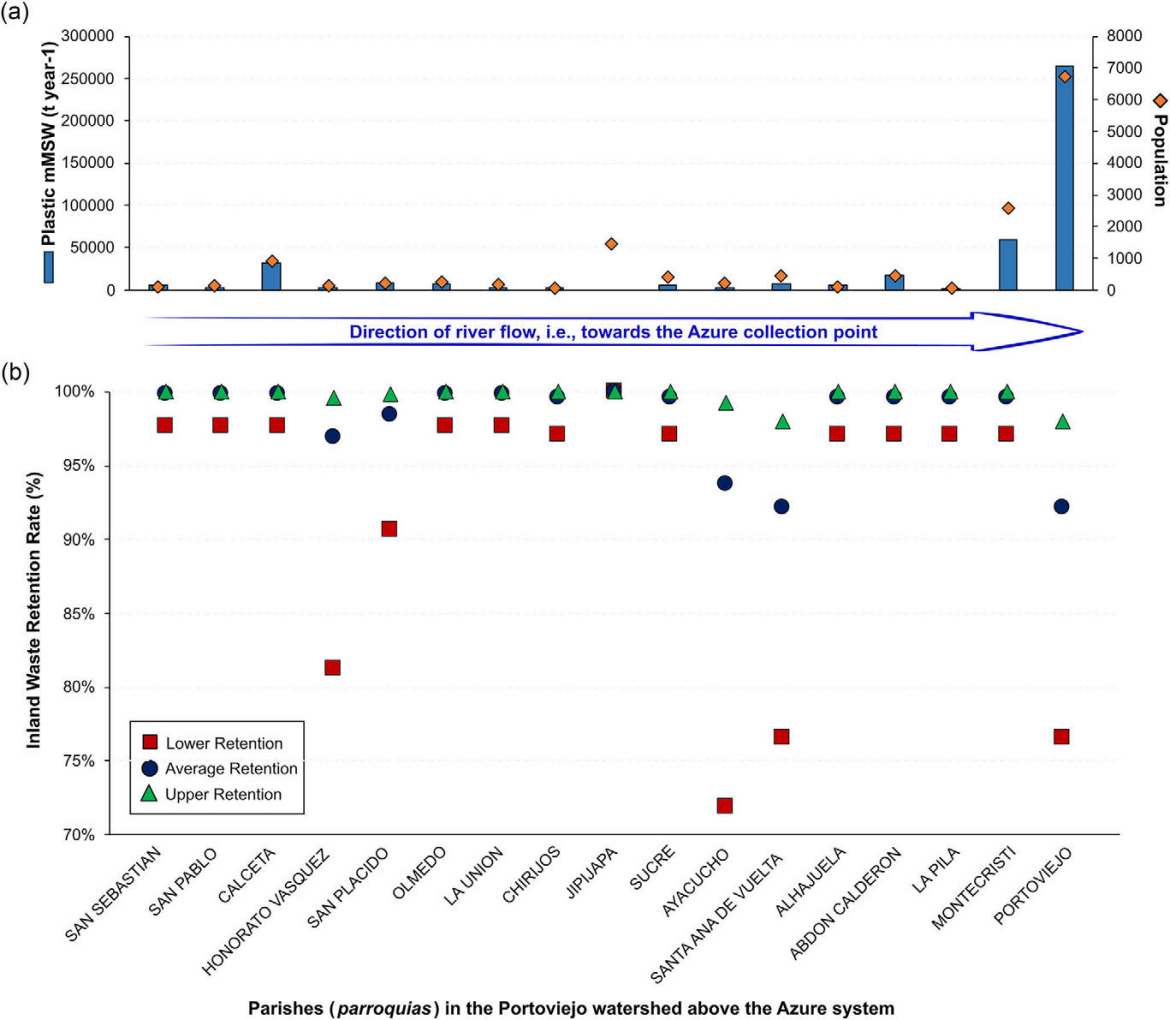
(a) Calculated mismanaged municipal solid waste (mMSW, in t year^−1^) and population for 2010 provided by the National Institute of Statistics and Censuses (*Instituto Nacional de Estadística y Censos*, INEC); (b) modeled inland plastic waste retention rates (in %) on upper, average, and lower scenarios of inland plastic retention capacities for the parishes (*parroquias*) located above the Azure collection system. The values on (b) depict the relationship between plastic flowing toward the Azure collection point and the total amount of mismanaged plastic waste in each parish. Both (a) and (b) share the same *x-*axis containing the names of the parishes. Underlying data for this figure are available in Supporting information S2.

Population in the parishes in the Portoviejo watershed located up the Azure system varied from 2717 inhabitants in Chirijos to 252,248 inhabitants in the city of Portoviejo, while the calculated mismanaged plastic waste (QmMSWp) for the same parishes varied from 0 t year^−1^ in Jipijapa to 7059.5 t year^−1^ in Portoviejo (Figure 4a). Information on waste management showed that only the Jipijapa province has a sanitary landfill, while others have either open or controlled dumps without adequate management (in Portoviejo and San Sebastian), or inadequate waste collection coverage. Morán Plua and Naranjo López (2022) mentioned that the Jipijapa landfill is currently inadequately managed, with waste deposited in open spaces, but part is retained inland in open dumps located far from water bodies. A general lack of information regarding the location of most dumps in the water basin was found, so three scenarios of inland plastic waste retention were considered in Equation (1), as part of the anthropogenic barriers. The amount of plastic waste toward the ocean (pWtO) modeled for the entire Portoviejo watershed varied between 190.1 and 2381.3 t year^−1^, with 785.6 t year^−1^ on the average retention scenario (Table 3). When considering only those cities located upstream of the Azure barrier (Figure 4b), it was estimated that 148.8 t year^−1^ for the lower retention scenario, 593.8 t year^−1^ for the average scenario, and 1858.7 t year^−1^ for the upper scenario are transported through the river Portoviejo toward the ocean. These values can be translated to plastic emissions per capita, ranging from 0.19 to 2.48 kg person^−1^ year^−1^, with an average of 0.79 kg person^−1^ year^−1^ by all cities up to the Azure barrier. The parishes of Portoviejo, Montecristi, and Calceta were estimated to have the largest waste emission toward the Azure system, that is, lower waste inland retention in all three scenarios evaluated (Figure 4b).

**TABLE 3:** Estimation of plastic waste-to-ocean generation for 1 year in each parish (*parroquia*) in the Portoviejo watershed.

		Population (INEC 2022 Census)			Total MSW (t year^−1^)	Plastic fraction (t year^−1^)	Formally recovered (t year^−1^)	Total plastic mismanaged (t year^−1^)	pWtO (t year^−1^)			Retention (%)		
Province (*Cantón*)	City (*Parroquia*)	Urban	Rural	Total					U	A	L	U	A	L
24 De Mayo	Sucre	6607	9038	15645	1541.8	172.1	3.2	168.9	4.9	0.7	0.0	97	100	100
Bolivar	Calceta	20011	14691	34702	7599.7	848.1	15.7	832.4	19.5	1.3	0.0	98	100	100
Jaramijo	Jaramijo	28397	1362	29759	9558.6	1066.7	19.7	1047.0	30.7	4.1	0.0	97	100	100
Jipijapa	Jipijapa	45382	9257	54639	19145.5	0.0	0.0	0.0	0.0	0.0	0.0	100	100	100
Junin	Junin	7332	14992	22324	6029.7	672.9	12.4	660.5	15.5	1.0	0.0	98	100	100
Montecristi	La Pila	0	3079	3079	460.8	51.4	1.0	50.5	1.5	0.2	0.0	97	100	100
	Montecristi	71066	25792	96858	14494.8	1617.6	29.9	1587.7	46.5	6.3	0.0	97	100	100
Olmedo	Olmedo	2684	7406	10090	1804.6	201.4	3.7	197.7	4.6	0.3	0.0	98	100	100
Pichincha	San Sebastian	0	4787	4787	1310.4	146.2	2.7	143.5	3.4	0.2	0.0	98	100	100
Portoviejo	San Placido	0	8180	8180	2090.0	233.2	4.3	228.9	21.5	3.6	0.5	91	98	100
	Abdon Calderon	0	16848	16848	4304.7	480.4	8.9	471.5	13.8	1.9	0.0	97	100	100
	Alhajuela	0	4714	4714	1204.4	134.4	2.5	131.9	3.9	0.5	0.0	97	100	100
	Chirijos	0	2717	2717	694.2	77.5	1.4	76.0	2.2	0.3	0.0	97	100	100
	Crucita	0	16997	16997	4342.7	484.6	9.0	475.7	167.3	75.0	19.0	65	84	96
	Portoviejo	244129	8119	252248	64449.4	7192.5	133.1	7059.5	1655.0	556.6	140.8	77	92	98
	Pueblo Nuevo	0	4181	4181	1068.2	119.2	2.2	117.0	3.4	0.5	0.0	97	100	100
	Rio Chico	0	7838	7838	2002.6	223.5	4.1	219.4	6.4	0.9	0.0	97	100	100
Rocafuerte	Rocafuerte	11848	30840	42688	12309.1	1373.7	25.4	1348.3	39.5	5.3	0.0	97	100	100
Santa Ana	Ayacucho	0	7934	7934	781.9	87.3	1.6	85.6	24.1	5.4	0.7	72	94	99
	Honorato Vasquez	0	5813	5813	572.9	63.9	1.2	62.7	11.8	2.0	0.3	81	97	100
	La Union	0	7200	7200	709.6	79.2	1.5	77.7	1.8	0.1	0.0	98	100	100
	San Pablo	0	6009	6009	592.2	66.1	1.2	64.9	1.5	0.1	0.0	98	100	100
	Santa Ana De Vuelta	10404	6476	16880	1663.5	185.6	3.4	182.2	42.7	14.4	3.6	77	92	98
Sucre	Charapoto	0	23361	23361	5798.2	647.1	12.0	635.1	223.3	100.1	25.3	65	84	96
Tosagua	La Estancilla	7332	14992	22324	4726.0	527.4	9.8	517.7	15.2	2.0	0.0	97	100	100
	Tosagua	11317	19992	31309	6628.1	739.7	13.7	726.0	21.3	2.9	0.0	97	100	100

*Note*: The table is organized in alphabetical order of province (*cantón*).

Abbreviations: U: upper scenario; A: average scenario; L: lower scenario.

## DISCUSSION

This study reports on how the Azure system, a manmade floating barrier, can be used to gather observational data on anthropogenic litter from a river course, providing data for assessing root causes of mismanaged waste in a watershed. The results shown here for the litter collected with the Azure system in the Portoviejo River are consistent and representative for 2 years of operation, except March and April, for which the dataset is incomplete, as the system had limited operation in those months due to technology restrictions related to river conditions. Nevertheless, our findings show that the Portoviejo River is contaminated with anthropogenic litter from several sources, but mainly from domestic waste (Table 2), and that seasonal variations might be occurring in the Portoviejo watershed (Figure 3). The collected amounts of plastic waste are distinct from modeled mismanaged plastic riverine runoff (Figure 4 and Table 3), which highlights the gap seen in other methodologies and modeling studies related to SA rivers, where empirical data from SA systems were not considered (e.g., Hurley et al., 2023; Meijer et al., 2021; Strokal et al., 2023). Models expand on often limited field sampling capacities and help combine several influencing factors into a single framework. Field sampling provides empirical data from a real scenario at a given time, which represents ground truth information of contamination. It is therefore crucial to validate/calibrate models to help achieve estimates that are closer to the truth. Both approaches can be used to inform action and help prioritize mitigation measures. These aspects are discussed below.

When comparing empirical data from the Azure system (Figure 3) with other observational studies, the Portoviejo River is transporting less waste than the Danube River (126 t month^−1^) (Lechner et al., 2014), two exemplary rivers in Southern California, USA (630 t month^−1^) (Moore et al., 2011), and the River Seine (161 t month^−1^) (Gasperi et al., 2014). Differences could be attributed to the inclusion of smaller items (millimeter scale), which can be representative. For example, in the work by Moore et al. (2011), the smaller fraction (1–4.75 mm) represented 15.8% of the overall collected weight, that is, 4.8 t in 24 h. Also in that study, they covered a greater river depth than in the current study. Gasperi et al. (2014) used a large network of floating booms along the river, which potentially has a higher waste recovery capacity than the Azure. In addition, these rivers differ in socioeconomic and environmental aspects, for example, local climate (e.g., wind, precipitation), landscape (e.g., land use, terrain slope), and water basin size. The distance from waste source to the river and the coastline, and hydrological factors such as river flow velocity, water level, and discharge will also affect the potential for litter to be transported to the ocean (Meijer et al., 2021; van Emmerik et al., 2018; van Emmerik et al., 2022). Thus, empirical data will show a scenario that can only be fully interpreted together with site characteristics at a given time.

An extensive meta-analysis performed by Morales-Caselles et al. (2021) analyzed macrolitter globally, and a database of more than 12 million items was created from seven major aquatic environments, including river waters and riverbeds. The authors found that food containers and plastic bags (here assumed empty) prevail in item counts in rivers, while plastic caps and lids and glass bottles are the most common items in riverbeds. The composition of litter collected by the Azure system fits those results partially, as plastic bags were the main material collected (Table 2). Plastic bags are usually low-density materials that can float in water for longer periods of time and, therefore, be transported for longer distances, but this might not apply to the heavier plastic bags filled with domestic waste as collected by the Azure system.

At the Portoviejo water basin, lower precipitation values historically occur between July and November (Campos Cedeno et al., 2018), as seen in Table 1. In this study, we found higher quantities of waste during the dry season than during the rainy season (Figure 3), even though it has been shown that increased rainfall can mobilize litter from land into rivers (van Emmerik et al., 2019), especially in extreme flood events where river discharge is substantially increased (e.g., van Emmerik et al., 2023). More litter was collected during lower river flow velocity in our study site (Figure 3 and Table 1), but this may have been caused by the technical limitations of the Azure system during the highest flow/river depth, when remobilized waste could evade the Azure barrier. This could partially explain the higher values found in the dry months in the current study, but an in-depth correlation analysis using an uninterrupted dataset is needed to confirm this. In addition, plastic transport on land may depend on the combination of material type and terrain characteristics, and less on rain intensity alone (Mellink et al., 2024), while river discharge can be a poor predictor of riverine plastic transport alone (Roebroek et al., 2022; van Emmerik, de Lange et al., 2022). For the Portoviejo region, floods have historically occurred due to steep slopes and the presence of extensive floodplains (Guerrero et al., 2022), combined with highly variable annual rainfall (Mendoza Alava et al., 2022). These floodplains have been indicated as garbage deposit areas, which together with depressed plains and swamp areas might contribute to the occurrence of strong, sudden, and destructive floods (Sandoval Erazo et al., 2022).

Domestic waste deposition can be a significant source of litter to the riverine systems, as exemplified in studies in the United Kingdom (Williams & Simmons, 1999), Chile (Rech et al., 2015), and Germany (Kiessling et al., 2019). The practice of using shopping bags to dispose of domestic waste is common in South America and other regions of the world (e.g., Senturk & Dumludag, 2021). Accordingly, our findings show filled plastic bags as the main type of litter collected (Table 2), suggesting that illegal domestic waste dumping is a major source of river contamination at the Portoviejo water basin. This was consistently witnessed during sampling (personal communication, 2022) and corroborates recent surveys by Briones-Bravo and Castro-Piguave (2023) showing that most domestic waste in the region is not recycled or reused and mostly goes to uncontrolled incineration. This highlights the strong relationship between poor waste management and river contamination, which brings responsibility closer to the governance level.

In Ecuador, municipalities implement monitoring actions for effective waste management (Cifuentes et al., 2021). The institutional and infrastructure capacity varies between municipalities, reflecting waste disposal operation cost, for example, waste disposal in landfills can vary from 33.1 to 300.6 $ t^−1^ (INEC, 2020). In Manabi province, only 64% of the municipalities have a waste treatment and disposal system; most of the waste (43%) in 2020 went to controlled or uncontrolled dumps (16%). Ecuador, along with other countries in the region (e.g., Chile, Colombia, Mexico, and Peru), has strong regulatory laws; however, when analyzing infrastructure and innovation capacity regarding plastic waste management, Ecuador has one of the lowest scores (Cifuentes et al., 2021). The lack of an effective waste management system can influence the quantity of plastic that reaches the ocean, and better governance can contribute to the reduction of plastic pollution (Nyberg et al., 2023). Cleaning technologies like the Azure provide valuable primary data on actual waste and the different types being mismanaged, which points to the different sources of contamination in the study area that can then be targeted for waste management solutions. The reports generated also serve as awareness materials for local communities and policy-makers. Furthermore, it could also help alleviate pressure on the environment and promote the recovery of materials, while acting as a tool to monitor changes in the system and the effectiveness of interventions when analyzing data in the long term. Yet, such initiatives must not be used to push back efforts to source the region with improved waste management infrastructure, and a systematic approach is still needed to reduce the quantity of plastic reaching the river.

The use of mathematical models to estimate environmental scenarios of contamination is helpful to build upon the interpretations from empirical data by adding multiple factors into the same framework. Using the method of Ita-Nagy et al. (2022), this study estimated an annual emission of plastics between 145.8 and 1858.7 t per year transported by the Portoviejo River towards the ocean, before the Azure system location (Figure 4a and Table 3). The amount of plastic waste collected by the Azure system (5.7 t per year in 2021; 8.1 t per year in 2022) represents a fraction of this estimation. The difference between estimated waste generation in the watershed and observational data from the Azure can be attributed to several factors. First, environmental characteristics in upper areas of the watershed may prevent or delay waste transport, resulting in low waste recovery at the Azure collection point. Examples are differences in precipitation or river flows, or landscape features such as floodplains and vegetation, and manmade barriers such as dams, hydroelectric plants, drainage systems and irrigation canals that trap and retain litter along the river course, even if temporarily (van Emmerik et al., 2022). Other physical features that may retain and delay the movement of waste are bends in the Portoviejo River course (Newbould et al., 2021) (Figure 1), although they may account for only a small amount of waste. Also, the model accounts for plastic emissions over the entire year, while our empirical data lacks data points in the rainy season. Regarding how to refine model calculations, incineration should ideally be accounted for as a new factor. Illegal and uncontrolled domestic waste incineration is recurrent in the area (personal communication and observation), which would then prevent the waste from reaching the river course, but unfortunately, there is no official data for the region under study. A well-known barrier in the Portoviejo watershed (i.e., the Poza Honda dam) was accounted for in the predictions of mismanaged waste, but there is a lack of official information regarding additional barriers contributing to waste retention, which directly affects factor *f*_ca_ in the model. Most parishes in the Portoviejo water basin had high inland plastic retention rates (all over 70% retention, see Figure 4), which relates to either the distance of the cities from the main river course (Figure 1) or potential waste accumulation zones inland. In addition, the model used official data on waste production per capita and plastic recycling rates for organization levels higher than parishes, that is, country or province level, so their accuracy cannot be verified at the parish level. Therefore, further investigations are needed on these, as well as adding river morphological features such as bends as parameters in future riverine mismanaged waste estimates.

The Azure system was removed from the river for short periods during strong precipitation events that may wash and accelerate the transport of waste in the river, which was not accounted for in this study. These events happened mostly during March or April. Considering the different buoyancy behaviors of anthropogenic waste (GESAMP et al., 2015), a fraction of waste materials could be passing underneath the Azure barrier, especially when river depth is very high (e.g., when the Azure can only cover 18% of total river depth). The differences in litter buoyancy might be contributing to the disparity between collected and modeled data, but this is also not currently considered in our plastic riverine transport estimation model. In general, high-density plastics will tend to sink more easily than low-density ones; however, low-density plastics with large surface area can get material accretion, such as mud or biofouling, making them heavier and more likely to remain in the lower part of the river or to sink (van Emmerik & Schwarz, 2020). At the same time, items related to domestic waste, such as filled plastic bags and bottles, can trap air inside them and therefore be highly buoyant in water, despite the polymer type.

A study by Schuyler et al. (2021) found a stronger correlation of plastic contamination with physical/environmental variables, infrastructure, and socioeconomics than with population density, which is commonly used as a main determining factor. For example, Meijer et al. (2021) found that when the land around the river is mainly artificial, that is, built land, the nearest river is likely to emit more plastic waste into the ocean than when the land is dominated by cultivated areas, even if the water basin and the waste generation are much bigger. At the Portoviejo basin, land use is dominated by arable land and plantations, while constructed areas are limited to scarce urban areas (Nguyen et al., 2017) (Figure 1). Waste generated in the basin might have lower mobility via river systems than in areas with larger constructed land. This raises a hypothesis that litter might be accumulating instead in the soil. According to our sampling data, the Portoviejo River transported annually at least three orders of magnitude less plastics than rivers in the top global 50 for annual plastic emission to the oceans which varied from 2.2 × 10^3^ to 1 × 10^6^ t per year according to estimates by Meijer et al. (2021), but it can reach a similar magnitude if looking at the presented mismanaged waste predictions (up to 2.3 × 10^3^ t year^−1^ in the lower retention scenario). However, the model by Meijer et al. (2021) includes environmental factors related to waste mobilization (e.g., precipitation and wind), slope, and river discharge, which are not accounted for in the model by Ita-Nagy et al. (2022) used here. On the other hand, Meijer et al. (2021) do not include any South American rivers in their model calibration or validation. Besides, the sampling point in this study is situated around 25 km from the Portoviejo river mouth (Figure 1), so actual emissions to the Pacific Ocean are likely to differ. Likewise, current models might be overestimating river waste exports as discussed by Weiss et al. (2021). Both observational data from South America and watershed-specific features might be decisive for improving the overall accuracy of global estimates.

## CONCLUSIONS

Overall, 2 years of monitoring anthropogenic litter in the Portoviejo River suggest direct deposition of domestic waste into the Portoviejo water basin as a major factor contributing to waste runoff. Both local climate and socio-economic factors influenced riverine anthropogenic waste, especially in water basins where waste management is faulty. Despite current limitations with the Azure system data collection, the barrier prevented 13.8 t of anthropogenic waste from reaching the ocean. In contrast, our model for predicting mismanaged waste reaching the Azure system in the Portoviejo River estimated an average of 445.4 t of plastic per year. This discrepancy might be explained by the limited information on factors affecting waste transport in the region, which would prevent the model from giving accurate estimates. These factors include geographical features, possible barriers to waste transport along the river course that were unaccounted for, and illegal incineration of domestic waste. Our findings highlight the urgent need for more observational data from systematic field sampling strategies and the need for applied systems that can measure anthropogenic litter continuously, including during high rainfall events which can be critical days in terms of waste mobilization. Modeling tools can expand on sampling capacities by combining influencing factors to estimate riverine waste runoff. Both approaches can be used to inform action and help prioritize mitigation measures by providing reliable data on riverine waste to guide mitigation measures in the Global South.

## Supplementary Information

**Supporting Information S1**: jiec70093-sup-0001-SuppMat.docx - This supporting information provides detailed methodology on the study area, hydrological and meteorological data collection, empirical data analysis, and details on the calculated parameters for plastic ocean-to-waste model calculations and where to find the data used for calculations. It also contains one supplementary figure (PCA analysis of litter categories among sampling months and years), and three supplementary tables containing the parameters of a two-way ANOVA test, the results of a SIMPER analysis, and the outputs of one- and two-way PERMANOVA tests.

**Supporting Information S2**: jiec70093-sup-0002-SuppMat.xlxs - This supporting information provides plastic ocean-to-waste model calculations, its parameters and data used for the Portoviejo watershed. Data includes population, waste production, waste management, and recycling rates for each parish located inside the Portoviejo watershed.

## Data Availability

The research data supporting this publication can be openly found at the Environmental Information Data Centre at https://doi.org/10.5285/e78e1cef-e30b-4313-8733-c03e1a7b7b2f and https://doi.org/10.5285/6d2ad65f-1a8f-4819-892b-1b4de8d0d7c2. The Portoviejo urban areas shapefiles are openly available at: http://ide.ambiente.gob.ec:8080/mapainteractivo/. This work is licensed under Creative Commons Attribution Licence CC BY (only). To view a copy of this license, please visit http://creativecommons.org/licenses/by/4.0/.

## References

[CR1] Anderson, A., Andrady, A., Arthur, C., Baker, J., Bouwman, H., Gall, S., Hidalgo-Ruz, Valeria, K., Angela, L., Lavender, K., Leslie, H., Kershaw, P., Pahl, S., Potemra, J., Ryan, P., Shim, W. J., Thompson, R., Takada, H., Wyles, K., GESAMP. (2015). *Sources, fate and effects of microplastics in the marine environment: A global assessment*. http://ec.europa.eu/environment/marine/good-environmental-status/descriptor-10/pdf/GESAMP_microplasticsfullstudy.pdf

[CR2] Bhat, R. A., Singh, D. V., Qadri, H., Dar, G. H., Dervash, M. A., Bhat, S. A., Unal, B. T., Ozturk, M., Hakeem, K. R., & Yousaf, B. (2022). Vulnerability of municipal solid waste: An emerging threat to aquatic ecosystems. Chemosphere, 287(3), 132223. 10.1016/j.chemosphere.2021.132223 34537459 10.1016/j.chemosphere.2021.132223

[CR3] Briones-Bravo, M. J., & Castro-Piguave, C. A. (2023). Optimización económica en la generación de los desechos orgánicos e inorgánicos en el cantón Portoviejo. MQRInvestigar, 7(3), 2629–2645. 10.56048/MQR20225.7.3.2023.2629-2645

[CR4] Campos Cedeno, A. F., Mendoza Alava, J. O., Sinichenko, E. K., & Gritsuk, I. I. (2018). Influence of the El Niño phenomena on the climate change of the Ecuadorian coast. RUDN Journal of Engineering Researches, 19(4), 513–523. 10.22363/2312-8143-2018-19-4-513-523

[CR5] Cifuentes, L. A., Cerda Gho, V., Bohaud Ausset, A.-L., Alarcón González, M. T., Cabrera Castro, C., Parra Arias, J. L., López Gómez, I., Ochoa Herrera, V., Cevallos, D. F., Flores Rendón, C., Scheel Mayenberger, C., Cantú Garza, A., Galarza Contreras, E., & Galarza Contreras, C. (2021). *Gestión sostenible de plásticos: análisis regulatorio y técnico en el marco de la iniciativa de economía circular en la Alianza del Pacífico y Ecuador*. 10.18235/0003633

[CR6] Cuenca Zambrano, K., & Pacheco Gil, H. (2021). Vegetation dynamics and climate variability in the Portoviejo river basin. Revista de La Facultad de Agronomía, Universidad Del Zulia, 38(3), 662–680. 10.47280//revfacagron(luz).v38.n3.11

[CR7] Diéguez-Santana, K., Sarduy-Pereira, L. B., & De Decker, M. (2021). Characterization and quantification of municipal solid waste in Fátima, Ecuadorian Amazon Parish. Journal of Environmental Treatment Techniques, 9(2), 392–401. 10.47277/JETT/9(2)401

[CR8] Eriksen, M., Cowger, W., Erdle, L. M., Coffin, S., Villarrubia-Gómez, P., Moore, C. J., Carpenter, E. J., Day, R. H., Thiel, M., & Wilcox, C. (2023). A growing plastic smog, now estimated to be over 170 trillion plastic particles afloat in the world's oceans—Urgent solutions required. PLoS ONE, 18(3), e0281596. 10.1371/journal.pone.0281596 36888681 10.1371/journal.pone.0281596 PMC9994742

[CR9] Falk-Andersson, J., Rognerud, I., De Frond, H., Leone, G., Karasik, R., Diana, Z., Dijkstra, H., Ammendolia, J., Eriksen, M., Utz, R., Walker, T. R., & Fürst, K. (2023). Cleaning up without messing up: Maximizing the benefits of plastic clean-up technologies through new regulatory approaches. Environmental Science & Technology, 57(36), 13304–13312. 10.1021/acs.est.3c01885 37638638 10.1021/acs.est.3c01885 PMC10501118

[CR10] Gasperi, J., Dris, R., Bonin, T., Rocher, V., & Tassin, B. (2014). Assessment of floating plastic debris in surface water along the Seine River. Environmental Pollution, 195, 163–166. 10.1016/j.envpol.2014.09.001 25240189 10.1016/j.envpol.2014.09.001

[CR11] Guerrero, E. E. B., Hidrovo, E. X. V., Menéndez, E. A. M., Zambrano, X. H. V., & Mata, W. J. M. (2022). Geomorphological and anthropogenic controls in differentiated extreme hydrological responses of micro-watersheds of the Manabi coast (Ecuador). In G. B. dos Santos, M. F. Felippe, & R. M. Neto (Eds.), XIII Sinageo Geomorfologia: Complexidade e interescalaridade da paisagem. Câmara Brasileira do Livro.

[CR12] Hammer, Ø., Harper, D. A. T., & Ryan, P. D. (2001). PAST: Paleontological Statistics software package for education and data analysis (3.21). Paleontologia Electronica, 4(1), 4, 1-9. https://palaeo-electronica.org/2001_1/past/issue1_01.htm

[CR13] Hatje, V., Rayfuse, R., Polejack, P., Goddard, C., Jiang, C., Jones, D., Faloutsos, D., Fiedler, H., Akrofi, J., Sheps, K., Leung, K., Pinheiro, L. M., Pradhan, M., Castrillejo, M., Bustamante, P., Kershaw, P., Zitoun, R., Silva, S., & Kiefer, T. (2024). Ocean Decade Vision 2030 White Papers—Challenge 1: Understand and beat marine pollution. The Ocean Decade Series, 51_1, 1–33. 10.25607/6m86-s908

[CR14] Hidalgo, J., Amaya, J., Jervis, F., & Moreira, C. (2019). Influence of socio-economic factors on household solid waste (HSW) generation of the city of Guayaquil, Ecuador. *Proceedings of the LACCEI International Multi-Conference for Engineering, Education and Technology*, *2019-July*. 10.18687/LACCEI2019.1.1.24

[CR15] Hurley, R., Braaten, H. F. V., Nizzetto, L., Steindal, E. H., Lin, Y., Clayer, F., van Emmerik, T., Buenaventura, N. T., Eidsvoll, D. P., Økelsrud, A., Norling, M., Adam, H. N., & Olsen, M. (2023). Measuring riverine macroplastic: Methods, harmonisation, and quality control. Water Research, 235, Article 119902. 10.1016/j.watres.2023.119902 36989801 10.1016/j.watres.2023.119902

[CR16] IFRC. (2023). *OPERATIONAL UPDATE Ecuador: Floods + earthquake-March 2023*. https://www.gestionderiesgos.gob.ec/wp-content/uploads/downloads/2023/06/SITREP-Nro-21-Esmeral

[CR17] INEC. (2020). *Municipales 2020*. https://www.ecuadorencifras.gob.ec/gad-municipales-2020/

[CR18] Ita-Nagy, D., Vázquez-Rowe, I., & Kahhat, R. (2022). Developing a methodology to quantify mismanaged plastic waste entering the ocean in coastal countries. Journal of Industrial Ecology, 26(6), 2108–2122. 10.1111/jiec.13349 42023063 10.1111/jiec.13349 PMC13098952

[CR19] Kiessling, T., Knickmeier, K., Kruse, K., Brennecke, D., Nauendorf, A., & Thiel, M. (2019). Plastic Pirates sample litter at rivers in Germany—Riverside litter and litter sources estimated by schoolchildren. Environmental Pollution, 245, 545–557. 10.1016/j.envpol.2018.11.025 30469125 10.1016/j.envpol.2018.11.025

[CR20] Lebreton, L., & Andrady, A. (2019). Future scenarios of global plastic waste generation and disposal. Palgrave Communications, 5(1), 10.1057/s41599-018-0212-7

[CR21] Lebreton, L. C. M., van der Zwet, J., Damsteeg, J., Slat, B., Andrady, A., & Reisser, J. (2017). River plastic emissions to the world's oceans. Nature Communications, 8(1), 15611. 10.1038/ncomms15611 10.1038/ncomms15611 PMC5467230 28589961

[CR22] Lechner, A., Keckeis, H., Lumesberger-Loisl, F., Zens, B., Krusch, R., Tritthart, M., Glas, M., & Schludermann, E. (2014). The Danube so colourful: A potpourri of plastic litter outnumbers fish larvae in Europe's second largest river. Environmental Pollution, 188, 177–181. 10.1016/j.envpol.2014.02.006 24602762 10.1016/j.envpol.2014.02.006 PMC3989055

[CR23] Lima, A. R. A., Silva, M. D., Possato, F. E., Ferreira, G. V. B., & Krelling, A. P. (2020). Plastic contamination in Brazilian freshwater and coastal environments: A source-to-sea transboundary approach. In F. Stock, G. Reifferscheid, N. Brennholt, & E. Kostianaia (Eds.), The handbook of environmental chemistry. Springer. 10.1007/698_2020_514

[CR24] Maalouf, A., & Mavropoulos, A. (2023). Re-assessing global municipal solid waste generation. Waste Management and Research, 41(4), 936–947. 10.1177/0734242X221074116 35075952 10.1177/0734242X221074116 PMC10114251

[CR25] Mai, L., Sun, X. F., Xia, L. L., Bao, L. J., Liu, L. Y., & Zeng, E. Y. (2020). Global riverine plastic outflows. Environmental Science and Technology, 54(16), 10049–10056. 10.1021/acs.est.0c02273 32700904 10.1021/acs.est.0c02273

[CR26] Margallo, M., Ziegler-Rodriguez, K., Vázquez-Rowe, I., Aldaco, R., Irabien, Á., & Kahhat, R. (2019). Enhancing waste management strategies in Latin America under a holistic environmental assessment perspective: A review for policy support. Science of the Total Environment, 689, 1255–1275. 10.1016/j.scitotenv.2019.06.393 31466164 10.1016/j.scitotenv.2019.06.393

[CR27] Meijer, L. J. J., van Emmerik, T., van der Ent, R., Schmidt, C., & Lebreton, L. (2021). More than 1000 rivers account for 80% of global riverine plastic emissions into the ocean. Science Advances, 7(18), eaaz5803. 10.1126/sciadv.aaz5803 33931460 10.1126/sciadv.aaz5803 PMC8087412

[CR28] Mellink, Y. A. M., van Emmerik, T. H. M., & Mani, T. (2024). Wind- and rain-driven macroplastic mobilization and transport on land. Scientific Reports, 14(1), 10.1038/s41598-024-53971-8 38365993 10.1038/s41598-024-53971-8 PMC10873394

[CR29] Mendoza Alava, J. O., Zambrano Xavier Horacio, V., Mendoza Cedeño, J. J., Sinichenko, E. K., Gritsuk, I. I., Alava, M. J., Xavier Horacioa, Z. V., & Cedeño, M. J. (2022). Phenomenological model of the intensity, duration and frequency of precipitation patterns for the Portoviejo river basin. Инженерные Исследования RUDN Journal of Engineering Research, 23(3), 246–253. 10.22363/2312-8143-2022-23-3-246-253

[CR30] Milliman, J. D., & Farnsworth, K. L. (2011). Runoff, erosion, and delivery to the coastal ocean. In River discharge to the coastal ocean (pp. 13–69). Cambridge University Press. 10.1017/cbo9780511781247.003

[CR31] Mmereki, D., Baldwin, A., & Li, B. (2016). A comparative analysis of solid waste management in developed, developing and lesser developed countries. Environmental Technology Reviews, 5(1), 120–141. 10.1080/21622515.2016.1259357

[CR32] Moore, C. J., Lattin, G. L., & Zellers, a. F. (2011). Quantity and type of plastic debris flowing from two urban rivers to coastal waters and beaches of Southern California. Revista de Gestão Costeira Integrada, 11(1), 65–73. 10.5894/rgci194

[CR33] Morales-Caselles, C., Viejo, J., Martí, E., González-Fernández, D., Pragnell-Raasch, H., González-Gordillo, J. I., Montero, E., Arroyo, G. M., Hanke, G., Salvo, V. S., Basurko, O. C., Mallos, N., Lebreton, L., Echevarría, F., van Emmerik, T., Duarte, C. M., Gálvez, J. A., van Sebille, E., Galgani, F., …Cózar, A. (2021). An inshore–offshore sorting system revealed from global classification of ocean litter. Nature Sustainability, 4(6), 484–493. 10.1038/s41893-021-00720-8

[CR34] Morán Plua, J. M., & Naranjo López, C. G. (2022). Afectación de las condiciones socio ambientales en la ciudad de Jipijapa a consecuencia del mal manejo de los residuos sólidos. MQRInvestigar, 6(4), 46–67. 10.56048/mqr20225.6.4.2022.46-67

[CR35] Nava, V., Chandra, S., Aherne, J., Alfonso, M. B., Antão-Geraldes, A. M., Attermeyer, K., Bao, R., Bartrons, M., Berger, S. A., Biernaczyk, M., Bissen, R., Brookes, J. D., Brown, D., Cañedo-Argüelles, M., Canle, M., Capelli, C., Carballeira, R., Cereijo, J. L., Chawchai, S., …, & Leoni, B. (2023). Plastic debris in lakes and reservoirs. Nature, 619(7969), 317–322. 10.1038/s41586-023-06168-4 37438590 10.1038/s41586-023-06168-4

[CR36] Newbould, R. A., Powell, D. M., & Whelan, M. J. (2021). Macroplastic debris transfer in rivers: A travel distance approach. Frontiers in Water, 3, 10.3389/frwa.2021.724596

[CR37] Nguyen, T. H. T., Boets, P., Lock, K., Forio, M. A. E., Van Echelpoel, W., Van Butsel, J., Utreras, J. A. D., Everaert, G., Granda, L. E. D., Hoang, T. H. T., & Goethals, P. L. M. (2017). Water quality related macroinvertebrate community responses to environmental gradients in the Portoviejo River (Ecuador). Annales de Limnologie, 53, 203–219. 10.1051/limn/2017007

[CR38] Nyberg, B., Harris, P. T., Kane, I., & Maes, T. (2023). Leaving a plastic legacy: Current and future scenarios for mismanaged plastic waste in rivers. Science of the Total Environment, 869, 10.1016/j.scitotenv.2023.161821 10.1016/j.scitotenv.2023.161821 36708835

[CR39] Orona-Návar, C., García-Morales, R., Loge, F. J., Mahlknecht, J., Aguilar-Hernández, I., & Ornelas-Soto, N. (2022). Microplastics in Latin America and the Caribbean: A review on current status and perspectives. Journal of Environmental Management, 309, Article 114698. 10.1016/j.jenvman.2022.114698 35183939 10.1016/j.jenvman.2022.114698

[CR40] OSPAR Commission. (2010). *Guideline for monitoring marine litter on the beaches in the OSPAR Maritime Area*. https://www.ospar.org

[CR41] Pinheiro, L. M., Agostini, V. O., Lima, A. R. A., Ward, R. D., & Pinho, G. L. L. (2021). The fate of plastic litter within estuarine compartments: An overview of current knowledge for the transboundary issue to guide future assessments. Environmental Pollution, 279, Article 116908. Elsevier Ltd. 10.1016/j.envpol.2021.116908 33774365 10.1016/j.envpol.2021.116908

[CR42] Rech, S., Macaya-Caquilpán, V., Pantoja, J. F., Rivadeneira, M. M., Campodónico, C. K., & Thiel, M. (2015). Sampling of riverine litter with citizen scientists—Findings and recommendations. Environmental Monitoring and Assessment, 187(6), 335. 10.1007/s10661-015-4473-y 25957193 10.1007/s10661-015-4473-y

[CR43] Roebroek, C. T. J., Laufkötter, C., González-Fernández, D., & van Emmerik, T. (2022). The quest for the missing plastics: Large uncertainties in river plastic export into the sea. Environmental Pollution, 312, 119948. 10.1016/j.envpol.2022.119948 36029903 10.1016/j.envpol.2022.119948

[CR44] Sandoval Erazo, W., Toulkeridis, T., Aguilar Ponce, A., Chiriboga, S. E., & Salazar, E. (2022). *Risk and vulnerability analysis of flood hazards in the Colón Parrish, Western Ecuador based on HEC-RAS Numerical Simulation* (pp. 245–260). 10.1007/978-3-031-08288-7_16

[CR45] Schmidt, C., Krauth, T., & Wagner, S. (2017). Export of plastic debris by rivers into the sea. Environmental Science and Technology, 51(21), 12246–12253. 10.1021/acs.est.7b02368 29019247 10.1021/acs.est.7b02368

[CR46] Schuyler, Q., Wilcox, C., Lawson, T. J., Ranatunga, R. R. M. K. P., Hu, C. S., & Hardesty, B. D. (2021). Human population density is a poor predictor of debris in the environment. Frontiers in Environmental Science, 9, 10.3389/fenvs.2021.583454

[CR47] SENAGUA. (2011). *No title*.

[CR48] Senturk, G., & Dumludag, D. (2021). An evaluation of the effect of plastic bag fee on consumer behavior: Case of Turkey. Waste Management, 120, 748–754. 10.1016/j.wasman.2020.10.042 33223247 10.1016/j.wasman.2020.10.042

[CR49] Siddiqua, A., Hahladakis, J. N., & Al-Attiya, W. A. K. A. (2022). An overview of the environmental pollution and health effects associated with waste landfilling and open dumping. Environmental Science and Pollution Research, 29(39), 58514–58536. 10.1007/s11356-022-21578-z 35778661 10.1007/s11356-022-21578-z PMC9399006

[CR50] Strokal, M., Vriend, P., Bak, M. P., Kroeze, C., van Wijnen, J., & van Emmerik, T. (2023). River export of macro- and microplastics to seas by sources worldwide. Nature Communications, 14(1), Article 4842. 10.1038/s41467-023-40501-9 10.1038/s41467-023-40501-9 PMC10415377 37563145

[CR51] UN Environment. (2018). *Waste management outlook for Latin America and the Caribbean*. In *United Nations Environment Programme*. https://wedocs.unep.org/bitstream/handle/20.500.11822/26448/Residuos_LAC_EN.pdf?sequence=2&isAllowed=y

[CR52] UNEP, GEF, & Danmarks Tekniske Universitet. (2018, October 23). *Share of plastic waste in municipal solid waste worldwide as of 2018, by region*. Statista.

[CR53] van Emmerik, T., de Lange, S., Frings, R., Schreyers, L., Aalderink, H., Leusink, J., Begemann, F., Hamers, E., Hauk, R., Janssens, N., Jansson, P., Joosse, N., Kelder, D., van der Kuijl, T., Lotcheris, R., Löhr, A., Mellink, Y., Pinto, R., Tasseron, P., …Vriend, P. (2022). Hydrology as a driver of floating river plastic transport. Earth's Future, 10(8), 10.1029/2022EF002811

[CR54] van Emmerik, T., Frings, R. M., Schreyers, L. J., Hauk, R., de Lange, S. I., & Mellink, Y. A. M. (2023). River plastic transport and deposition amplified by extreme flood. Nature Water, 1(6), 514–522. 10.1038/s44221-023-00092-7

[CR55] van Emmerik, T., Kieu-Le, T. C., Loozen, M., Oeveren, K. v., Strady, E., Bui, X. T., Egger, M., Gasperi, J., Lebreton, L., Nguyen, P. D., Schwarz, A., Slat, B., & Tassin, B. (2018). A methodology to characterize riverine macroplastic emission into the ocean. Frontiers in Marine Science, 5(OCT). 10.3389/fmars.2018.00372

[CR56] van Emmerik, T., Mellink, Y., Hauk, R., Waldschläger, K., & Schreyers, L. (2022). Rivers as plastic reservoirs. Frontiers in Water, 3, 10.3389/frwa.2021.786936

[CR57] van Emmerik, T., & Schwarz, A. (2020). Plastic debris in rivers. Wiley Interdisciplinary Reviews: Water, 7(1), 1–24. 10.1002/wat2.1398

[CR58] van Emmerik, T., Tramoy, R., van Calcar, C., Alligant, S., Treilles, R., Tassin, B., & Gasperi, J. (2019). Seine plastic debris transport tenfolded during increased river discharge. Frontiers in Marine Science, 6, 10.3389/fmars.2019.00642

[CR59] Weiss, L., Ludwig, W., Heussner, S., Canals, M., Ghiglione, J.-F., Estournel, C., Constant, M., & Kerhervé, P. (2021). The missing ocean plastic sink: Gone with the rivers. Science, 373(6550), 107–111. 10.1126/science.abe0290 34210886 10.1126/science.abe0290

[CR60] Williams, A. T., & Simmons, S. L. (1999). Sources of riverine litter: The River Taff, South Wales, UK. Water, Air, and Soil Pollution, 112(1/2), 197–216. 10.1023/A:1005000724803

