# Supplementary material for: Integrating technology and environmental data to predict mismanaged plastic waste in a watershed
Source: J Ind Ecol. 2025 Sep 4;29(5):1912–26. doi: 10.1111/jiec.70093 (PMC13275570; doi:10.1111/jiec.70093)
Supplement: Supplementary file 1 — Supporting Information S1: jiec70093-sup-0001-SuppMat.docx - This supporting information provides detailed methodology on the study area, hydrological and meteorological data collection, empirical data analysis, and details on the calculated parameters for plastic ocean-to-waste model calculations and where to find the data used for calculations. It also contains one supplementary figure (PCA analysis of litter categories among sampling months and years), and three supplementary tables containing the parameters of a two-way ANOVA test, the results of a SIMPER analysis, and the outputs of one- and two-way PERMANOVA tests. [file 44498_2025_2905030_MOESM1_ESM.docx]

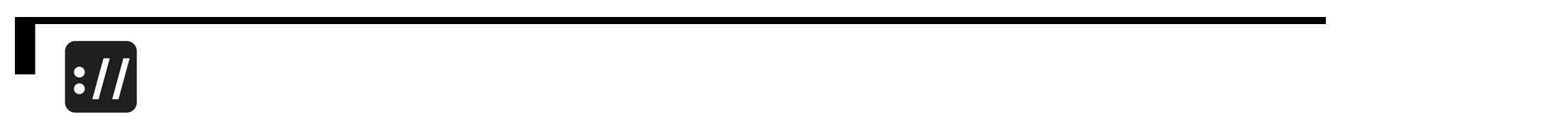


SUPPORTING INFORMATION FOR:

Pinheiro, L.M., Ita-Nagy, D., Hidalgo, D.G., Flor, D., Baquero, A.O., Becerra, N., Grønneberg, I., Vázquez-Rowe, I., Kahhat, R., Lewis, C. & Galloway, T.S. (2025) Integrating technology and environmental data to predict mismanaged plastic waste. *Journal of Industrial Ecology.*


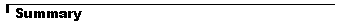


This supporting information provides detailed methodology on the study area, hydrological and meteorological data collection, empirical data analysis, and details on the calculated parameters for plastic ocean-to-waste model calculations and where to find the data used for calculations. It also contains one supplementary figure (PCA analysis of litter categories among sampling months and years), and three supplementary tables containing the parameters of a two-way ANOVA test, the results of a SIMPER analysis, and the outputs of one- and two-way PERMANOVA tests.


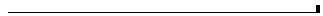


**Supplementary Methods**

*M1. Study Area*

The Portoviejo water basin drains an area of approximately 2,100 km^2^ and is considered a highly populated area: 64% in urban areas and 36% in rural areas (Guambo et al. 2022), but it is the main water source to over 600,000 people also considering the ones outside the watershed (Fernández et al. 2018; Chonlong et al. 2021). The total urban population inside the watershed is projected to be around 504,977 inhabitants in 2021 and quickly growing (INEC 2020). The water from this basin is essential to the main economic activity in the region, agriculture, which occupies over 50% of the economically active population (Chonlong et al. 2021). Other water uses in the region are domestic use, recreation, and others (Nguyen et al. 2017).

The elevation at the Portoviejo water basin reaches up to 600 m, and it is composed of two sub-basins corresponding to the Chico River and the Portoviejo River (Aguilar Ponce and Santiago Xavier 2018). For the present work it was only considered the second one since the Chico River joins the Portoviejo River at the lower basin, after the Azure system collection point which is described in the main manuscript.

The Poza Honda dam, located at the upper part of the Portoviejo watershed, covers an area of around 5 km^2^ and has 12 km in length (Utreras 2016). It was built between 1969 and 1971 with the purpose to provide irrigation and drinking water to the population (Utreras 2016). It has the capacity to hold 89 million m^3^ and the weir can release up to 875 m^3^ s^-1^ (personal communication). The area surrounding the dam has intensive agriculture and livestock, which led to eutrophication problems (Perez 2004). In addition, the watershed presents a variety of important ecosystems including the mountains in the higher part, the dry forest hills, the wildlife corridors in Rivers Portoviejo and Chico, and the estuarine region of La Boca with its riparian forest areas which provides habitat for several species and ecosystem services to the local population (Herrero 2007).

Annual rainfall in the higher region of the Portoviejo basin was found to vary between 1000-1400 mm, which is associated with the high terrain elevation (Guerrero et al. 2022), but values as high as 1921.50 mm year^-1^ were found by Erazo et al. (2022) for the Poza Honda dam region. This causes the Portoviejo River to be a permanent river, as it has input from higher regions where rainfall happens for longer (Montilla Pacheco and Pacheco Gil 2017). On the other hand, annual rainfall in the lower region varies between 300-600 mm (Guerrero et al. 2022). The average water discharge of the basin to the Pacific Ocean is of 11 m^3^ s^-1^, which happens in the region of La Boca (Figure 1) (Utreras 2016).

The Portoviejo River runs through the city of Portoviejo, which is the largest one in the province of Manabí with a population of approximately 280,000 inhabitants. It is the administrative and commercial centre for the province, and where productive economical relationships occur. There is one single open-air dump in the city of Portoviejo, located around 2.6 km from the Portoviejo River course and with an area of approximately 0.16 km ^2^, which has been historically seen as a contamination source and for that reason has been scheduled for closure in 2017 after 40 years of operation (Viera Torres et al. 2018). However, this dump remains operational (personal observation/communication).

*M2. Hydrological and Meteorological data*

Meteorological data of average temperature (°C), wind speed (m.s^-1^), and wind direction was provided by the Ecuatorian National Institute of Meteorology and Hydrology (Instituto Nacional de Meteorología e Hidrología - INAMHI), from station M1208 - La Teodomira, located at 1° 09' 51.0" S and 80° 23' 24.0" W, approximately 20 km from the Azure system up the Portoviejo River. Hydrological and meteorological data of precipitation (mm day^-1^), river flow velocity (m s^-1^), river discharge (m^3^ s^-1^), river depth (cm), and river width (m) were collected daily on weekdays on site at the Azure System. Precipitation data was retrieved daily from website accuweather.com for the location *Picoazá*. River flow velocity was calculated manually by dividing the distance covered by a reference buoy and the time taken for it travel the distance, measured with a stopwatch. This process was repeated three times and final average velocity was determined. River depth was measured twice a day (at 8am and 4.30pm) using a fixed limnimeter marked every 50cm. River width was measured daily with a measuring tape at a 90° angle from the river margins at 5 equidistant points, then the average width was calculated for daily reporting. River discharge was calculated by multiplying the value of measured river flow velocity by the measured river cross section area. River discharge and width were measured only from January 2021 until March 2022 for logistical reasons.

*M3. Litter Data Analysis*

Normality of data was tested using a Shapiro-Wilk test. As data assumptions were met (Shapiro-Wilk test p<0.001), a two-way Analysis of Variance (two-way ANOVA) was performed to check for significant differences in litter quantities between sampling months and sampling years (α=0.05), and a Tukey’s posthoc test was used to locate these differences. To check for differences in litter quantities between rainy (December-June) and dry seasons (July-November), a Welch F test was performed as variances were found unequal following a Levene’s test (p>0.05). In addition, a Similarity Percentage (SIMPER) analysis with a Bray-Curtis similarity measure was performed to assess how much each litter category contributes to the differences in composition (litter categories) between sampling months (February - December) and years (2021 and 2022), while an Analysis of Similarity (ANOSIM) test was performed to check for the overall significance of this difference. To visualize the distribution of litter categories among sampling months and years, a Principal Component Analysis was performed, and the significance of the resulting distribution was verified using a PERMANOVA with a Euclidean similarity index.

*M4. Population, Waste production per capita, waste management, and recycling rates*

Detailed explanation of factors and parameters used in our model for estimating plastic waste towards the ocean (pWtO, Equation 1) can be seen on the Table S1 adapted from Ita-Nagy et al. (2022). Data and calculated parameters used for plastic ocean-to-waste model calculations at the Portoviejo watershed can be found on the separate Excel file named Supporting_Information_SM2_LPinheiro. The factors *f* from Ita-Nagy et al. (2022) were adapted to the Portoviejo watershed characteristics where possible, as also seen on the Table SM2. The hard plastic recovery rate of 5.8% (considered as the only fraction recycled) used in factor *f*_sr_ was calculated as an average of values for the Portoviejo parishes provided by the Solid Waste Management (Gestión de Resíduos Sólidos, GRS) for 2021 in Ecuador. The waste retention values (%) attributed to the three scenarios (upper, average, lower retention) were the same used by Ita-Nagy et al. (2022), chosen based on either previous studies or arbitrarily due to lack of data. Parishes distances to main river were calculated from geographical data (shapefiles) provided by INEC, by measuring the distance of the geographical center of each parish to the nearest point in the Portoviejo River.

**Table M1.** Coefficients of waste dissipation proposed by Ita-Nagy et al. (2022) for factors affecting waste transport in a water basin. *f*_cl_, coastline factor; *f*_ci_, inter-basin factor; *f*_cw_, closeness to main river factor; *f*_rs_, river seasonality factor; *f*_ca_, connection to an anthropogenic or natural barrier factor (e.g. the Azure System); *f*_sr_, scavengers’ recovery factor; *r*_PET_, fraction of PET bottles in waste stream.

|  |  | **Coefficients of waste dissipation** | | |
| --- | --- | --- | --- | --- |
| **Factor** | **Characteristic** | **Upper** | **Average** | **Lower** |
| *f*_cl_ | 0 - 0.1 km | 1.00 | 0.80 | 0.60 |
| *f*_ci_ | > 0.1 km | 0.20 | 0.10 | 0.05 |
| *f*_cw_ | 0 - 0.1 km to main river | 1.00 | 0.80 | 0.60 |
|  | > 0.1 - 1 km to main river | | | |
|  | Lower course | 0.80 | 0.60 | 0.40 |
|  | Middle course | 0.60 | 0.40 | 0.20 |
|  | Upper course | 0.40 | 0.40 | 0.10 |
|  | > 1 – 5 km to main river |  |  |  |
|  | Lower course | 0.60 | 0.40 | 0.20 |
|  | Middle course | 0.40 | 0.40 | 0.10 |
|  | Upper course | 0.20 | 0.10 | 0.05 |
|  | > 5km to main river | 0.05 | 0.01 | 0.00 |
| *f*_rs_ | Intermittent | 1.00 | 0.80 | 0.60 |
|  | Perennial | 1.00 | 1.00 | 1.00 |
| *f*_ca_ | Hydropower (run of the river) | 0.80 | 0.40 | 0.20 |
|  | Hydropower (impoundment) | 0.20 | 0.10 | 0.05 |
|  | Reservoirs, dams | 0.80 | 0.40 | 0.20 |
|  | Clean up barrier (Azure System) | 0.80 | 0.40 | 0.20 |
|  | No barrier | 1.00 | 1.00 | 1.00 |
| *f*_sr_ | Informal PET recovery | *r*_PET_*0.40 | *r*_PET_*0.25 | *r_P_*_ET_*0.05 |

**Supplementary Figures**


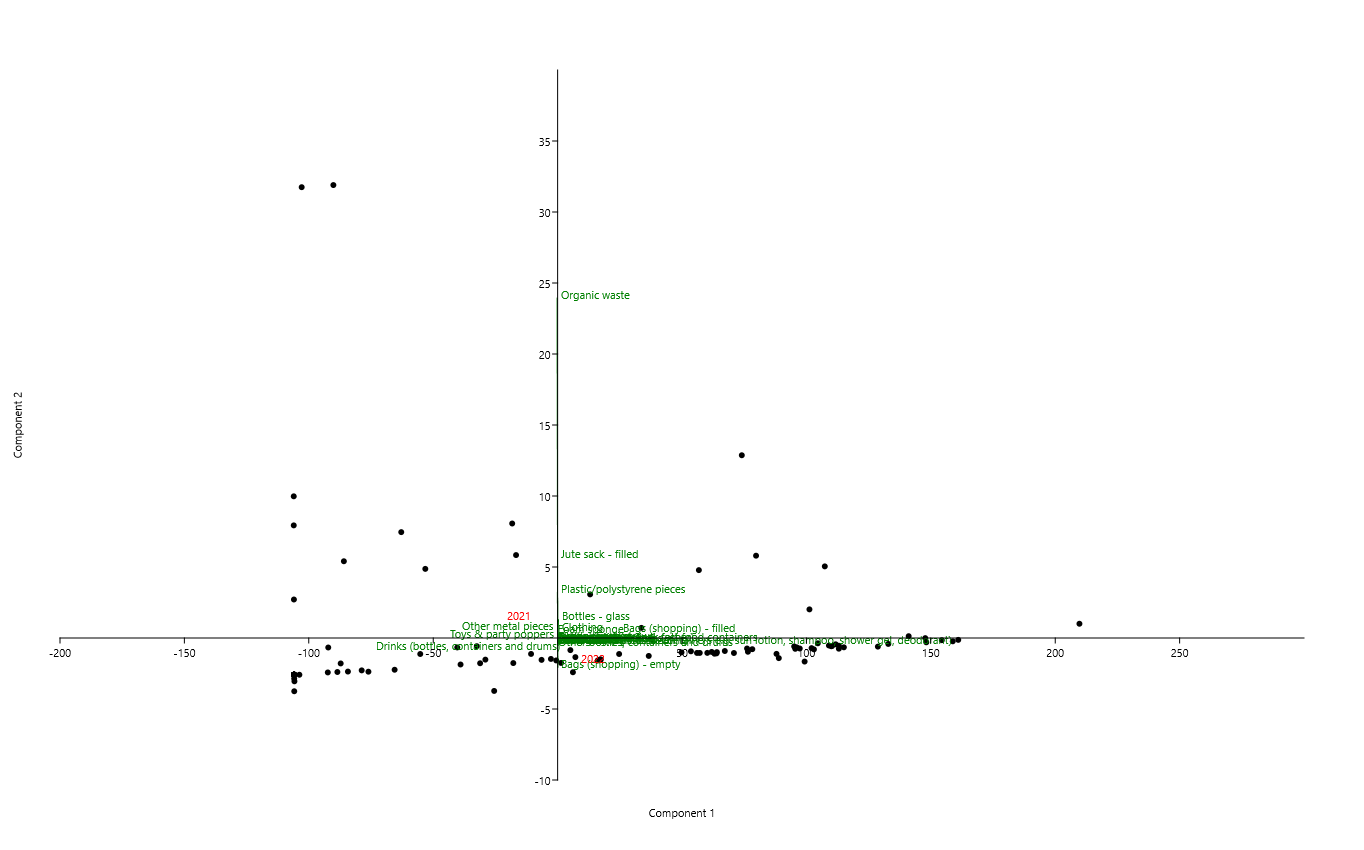


**Figure S1.** Principal Component Analysis (PCA) of the litter categories collected with the Azure system at the Portoviejo River among sampling months and years (February 2021 - December 2022). Component 1: 99.25% variance; Component 2: 0.31281% variance.

**Supplementary Tables**

**Table S1.** Parameters of the two-way Analysis of Variance (ANOVA) test for significant differences in litter quantities between sampling months and sampling years. Stars (*) indicate significance considering α=0.05.

|  | **Sum of sqrs** | **df** | **Mean square** | **F** | **p (same)** |
| --- | --- | --- | --- | --- | --- |
| **Year:** | 947.496 | 1 | 947.496 | 3.019 | 8.58E-02 |
| **Month:** | 9500.27 | 10 | 950.027 | 3.027 | 2.46E-03* |
| **Interaction:** | 618.406 | 10 | 61.8406 | 0.197 | 9.96E-01 |
| **Within:** | 27621.5 | 88 | 313.881 |  |  |
| **Total:** | 38687.7 | 109 |  |  |  |

**Table S2.** Results of Similarity Percentage Analysis (SIMPER) regarding weight (kg) of each litter category (OSPAR, 2010) collected from February - December on years 2021 and 2022 with the Azure system at the Portoviejo River.

| **OSPAR Litter Category** | **Av. Dissim.** | **Contrib. %** | **Cumulative %** | **Mean 2021** | **Mean 2022** |
| --- | --- | --- | --- | --- | --- |
| Bags (shopping) – filled with domestic waste | 49.51 | 77.52 | 77.52 | 90.4 | 119 |
| Organic waste | 2.626 | 4.112 | 81.63 | 3.14 | 0.126 |
| Drinks (bottles, containers, and drums) | 1.876 | 2.938 | 84.57 | 2.04 | 1.96 |
| Bottles - glass | 1.619 | 2.535 | 87.11 | 2.22 | 2.78 |
| Bags (shopping) - empty | 1.418 | 2.221 | 89.33 | 0.456 | 2.18 |
| Other bottles, containers, and drums | 1.131 | 1.771 | 91.1 | 0.507 | 1.56 |
| Plastic/polystyrene pieces | 1.009 | 1.579 | 92.68 | 1.24 | 0.0456 |
| Clothing | 0.7611 | 1.192 | 93.87 | 1.04 | 1.3 |
| Food containers incl. fast food containers | 0.5831 | 0.913 | 94.78 | 0.715 | 1.12 |
| Packaging, plastic sheeting | 0.4844 | 0.7586 | 95.54 | 0.268 | 0.654 |
| Foam sponge | 0.4669 | 0.7312 | 96.27 | 0.334 | 0.445 |
| Cosmetics (bottles & containers e.g., sun lotion, shampoo, shower gel, deodorant) | 0.4592 | 0.7191 | 96.99 | 0.597 | 0.638 |
| Cartons e.g., Tetrapak (other) | 0.392 | 0.6138 | 97.61 | 0.504 | 0.517 |
| Jute sack - filled | 0.3034 | 0.4751 | 98.08 | 0.389 | 0 |
| Other textiles | 0.2293 | 0.359 | 98.44 | 0.311 | 0.263 |
| Toys & party poppers | 0.2103 | 0.3293 | 98.77 | 0.0511 | 0.305 |
| Plastic sheeting | 0.1668 | 0.2613 | 99.03 | 0.181 | 0.309 |
| Food cans | 0.08988 | 0.1407 | 99.17 | 0.0991 | 0 |
| Cutlery/trays/straws | 0.07742 | 0.1212 | 99.29 | 0 | 0.0382 |
| Other metal pieces | 0.05965 | 0.09341 | 99.39 | 0 | 0.122 |
| Animal waste | 0.05928 | 0.09282 | 99.48 | 0.0536 | 0.0858 |
| Caps/lids | 0.05231 | 0.08191 | 99.56 | 0.153 | 0 |
| Drink cans | 0.05128 | 0.0803 | 99.64 | 0.0352 | 0.0398 |
| Other sanitary items | 0.048 | 0.07517 | 99.72 | 0.0195 | 0.0473 |
| Light bulbs/tubes | 0.04733 | 0.07411 | 99.79 | 0.0564 | 0 |
| Aerosol/spray cans | 0.04095 | 0.06412 | 99.85 | 0.008 | 0.0596 |
| Other | 0.0394 | 0.0617 | 99.92 | 0.0836 | 0 |
| Face mask | 0.0242 | 0.0379 | 99.95 | 0.026 | 0.0327 |
| Hard hats | 0.01761 | 0.02757 | 99.98 | 0.04 | 0 |
| Paraffin or wax pieces | 0.007172 | 0.01123 | 99.99 | 0.00927 | 0 |
| Cardboard | 0.004905 | 0.00768 | 100 | 0.00818 | 0 |
| Plastic tubes | 0 | 0 | 100 | 0 | 0 |
| Cups | 0 | 0 | 100 | 0 | 0 |

**Table S3.** Outputs of a one- and two-way permutational multivariate analysis of variance (PERMANOVA) using a Euclidean similarity index performed with 33 principal components from a Principal Component Analysis (PCA) of the litter categories collected with the Azure system at the Portoviejo River among sampling months and years (February 2021 to December 2022). Significances (p<0.05) are indicated in bold and a star (*).

| **Source** | | | | **Sum of sqrs** | | | | **df** | | **Mean square** | | | | **F** | | | **p** | |  |
| --- | --- | --- | --- | --- | --- | --- | --- | --- | --- | --- | --- | --- | --- | --- | --- | --- | --- | --- | --- |
| Year | | | | 25801.1 | | | | 1 | | 25801 | | | | 4.4207 | | | **3.70E-02*** | |  |
| Month | | | | 366749 | | | | 11 | | 33341 | | | | 5.7125 | | | **1.00E-04*** | |  |
| Interaction | | | | 30499.6 | | | | 11 | | 2772.7 | | | | 0.47506 | | | 8.70E-01 | |  |
| Residual | | | | 531117 | | | | 91 | | 5836.4 | | | |  | | |  | |  |
| Total | | | | 9.54E+05 | | | | 114 | |  | | | |  | | |  | |  |
| **YEARS: p-values** | | | | | | | | | | | | | | | | | | |  |
|  | 2021 | | | | | | | 2022 | | | | | | | | | | |  |
| 2021 |  | | | | | | | 8.20E-02 | | | | | | | | | | |  |
| 2022 | 8.20E-02 | | | | | | |  | | | | | | | | | | |  |
| **MONTHS: p-values** | | | | | | | | | | | | | | | | | | |  |
|  | | Feb | Mar | | Apr | May | Jun | | Jul | | Aug | Sep | Oct | | Nov | Dec | | Jan | |
| Feb | |  | 1.52E-01 | | 8.68E-01 | 8.20E-01 | 7.09E-01 | | 3.81E-01 | | 8.63E-02 | **6.80E-03*** | **1.52E-02*** | | 1.20E-01 | **1.50E-03*** | | 1.48E-01 | |
| Mar | | 1.52E-01 |  | | **4.58E-02*** | 6.48E-02 | **1.33E-02*** | | **4.70E-03*** | | **2.60E-03*** | **1.00E-04*** | **6.00E-04*** | | **2.30E-03*** | **1.00E-04*** | | **5.20E-03*** | |
| Apr | | 8.68E-01 | **4.58E-02*** | |  | 9.64E-01 | 5.10E-01 | | 2.14E-01 | | **3.17E-02*** | **3.00E-03*** | **5.30E-03*** | | **4.68E-02*** | **2.00E-04*** | | **4.98E-02*** | |
| May | | 8.20E-01 | 6.48E-02 | | 9.64E-01 |  | 4.59E-01 | | 1.92E-01 | | **3.08E-02*** | **1.70E-03*** | **5.00E-03*** | | **4.14E-02*** | **1.00E-04*** | | 5.26E-02 | |
| Jun | | 7.09E-01 | **1.33E-02*** | | 5.10E-01 | 4.59E-01 |  | | 5.36E-01 | | 8.97E-02 | **7.50E-03*** | **1.46E-02*** | | 1.45E-01 | **6.00E-04*** | | 1.41E-01 | |
| Jul | | 3.81E-01 | **4.70E-03*** | | 2.14E-01 | 1.92E-01 | 5.36E-01 | |  | | 2.29E-01 | **2.39E-02*** | 5.11E-02 | | 3.90E-01 | **6.00E-03*** | | 3.51E-01 | |
| Aug | | 8.63E-02 | **2.60E-03*** | | **3.17E-02*** | **3.08E-02*** | 8.97E-02 | | 2.29E-01 | |  | 3.92E-01 | 4.95E-01 | | 6.36E-01 | 2.12E-01 | | 8.20E-01 | |
| Sep | | **6.80E-03*** | **1.00E-04*** | | **3.00E-03*** | **1.70E-03*** | **7.50E-03*** | | **2.39E-02*** | | **3.92E-01** |  | 8.56E-01 | | 1.50E-01 | 7.18E-01 | | 3.14E-01 | |
| Oct | | **1.52E-02*** | **6.00E-04*** | | **5.30E-03*** | **5.00E-03*** | **1.46E-02*** | | 5.11E-02 | | 4.95E-01 | 8.56E-01 |  | | 2.27E-01 | 6.25E-01 | | 4.38E-01 | |
| Nov | | 1.20E-01 | **2.30E-03*** | | **4.68E-02*** | **4.14E-02*** | 1.45E-01 | | 3.90E-01 | | 6.36E-01 | 1.50E-01 | 2.27E-01 | |  | **4.23E-02*** | | 8.34E-01 | |
| Dec | | **1.50E-03*** | **1.00E-04*** | | **2.00E-04*** | **1.00E-04*** | **6.00E-04*** | | **6.00E-03*** | | 2.12E-01 | 7.18E-01 | 6.25E-01 | | **4.23E-02*** |  | | 1.19E-01 | |
| Jan | | 1.48E-01 | **5.20E-03*** | | **4.98E-02*** | 5.26E-02 | 1.41E-01 | | 3.51E-01 | | 8.20E-01 | 3.14E-01 | 4.38E-01 | | 8.34E-01 | 1.19E-01 | |  | |

**Supplementary References**

Aguilar Ponce, R.A. and E.C. Santiago Xavier. 2018. ANÁLISIS DE VULNERABILIDAD A INUNDACIONES DE LA PARROQUIA COLÓN, CANTÓN PORTOVIEJO-MANABI. Sangolquí: Universidad de las Fuerzas Armadas.

Chonlong, J., H. Pacheco, J. Cesar, and M. Leal. 2021. ESTIMATION OF POTENTIAL GROUNDWATER ZONES IN THE PORTOVIEJO RIVER BASIN THROUGH HIERARCHICAL ANALYSIS BASED ON GIS AND REMOTE SENSING ABSTRACT. *Publicación Cuatrimestral* 6(1): 1–18. https://doi.org/10.33936/rev_bas_de_la_ciencia.v%vi%i.2648.

Fernández, L.S.Q., E.I. Kulich, and C.M. Gutiérrez. 2018. ESTUDIO DEL IMPACTO AMBIENTAL DEL VERTIMIENTO DE AGUAS RESIDUALES SOBRE LA CAPACIDAD DE AUTODEPURACIÓN DEL RÍO PORTOVIEJO, ECUADOR. *Centro Azúcar* 45(1): 73–83.

Guambo, G., J. Torres, and S. Quiroz. 2022. Historical analysis of water pollution in the Portoviejo River. *Minerva* 3(8): 54–60.

Guerrero, E.E.B., E.X.V. Hidrovo, E.A.M. Menéndez, X.H.V. Zambrano, and W.J.M. Mata. 2022. Geomorphological and anthropogenic controls in differentiated extreme hydrological responses of micro-watersheds of the Manabi coast (Ecuador). In *XIII Sinageo Geomorfologia: Complexidade e Interescalaridade Da Paisagem*, ed. by Gisele Barbosa dos Santos, Miguel Fernandes Felippe, and Roberto Marques Neto. São José dos Campos: Câmara Brasileira do Livro.

Herrero, J.A. 2007. Fajas Forestales Hidrorreguladoras. Situación e importancia. *Rev. Agricultura Orgánica* 1: 40–42.

INEC. 2020. Municipales 2020. https://www.ecuadorencifras.gob.ec/gad-municipales-2020/. Accessed December 21, 2023.

Ita-Nagy, D., I. Vázquez-Rowe, and R. Kahhat. 2022. Developing a methodology to quantify mismanaged plastic waste entering the ocean in coastal countries. *Journal of Industrial Ecology* 26(6): 2108–2122.

Montilla Pacheco, A.D.J. and H.A. Pacheco Gil. 2017. Comportamiento temporal y espacial del bosque ribereÑo en el curso bajo del rÍo portoviejo y la quebrada chilÁn, provincia de ManabÍ, Ecuador. *Revista Internacional de Contaminacion Ambiental* 33(1): 21–35.

Nguyen, T.H.T., P. Boets, K. Lock, M.A.E. Forio, W. Van Echelpoel, J. Van Butsel, J.A.D. Utreras, et al. 2017. Water quality related macroinvertebrate community responses to environmental gradients in the Portoviejo River (Ecuador). *Annales de Limnologie* 53: 203–219.

Perez, Q.F.H.W.Y. 2004. Study the Physical, Chemical and Biological of Eutrophication Process of Poza Honda Reservoir and The Impact on the Formation of Trihalomethanes in the Regional Water-Supply System of Poza Honda (In Spanish). Guayaquil: Universidad De Guayaquil .

Sandoval Erazo, W., T. Toulkeridis, A. Aguilar Ponce, S.E. Chiriboga, and E. Salazar. 2022. Risk and Vulnerability Analysis of Flood Hazards in the Colón Parrish, Western Ecuador Based on HEC-RAS Numerical Simulation. In , 245–260.

Utreras, J.A.D. 2016. Ecological assessment of Portoviejo river basin (Ecuador). Gent: Universiteit Gent.

Viera Torres, M., M.J. Merizalde Mora, L. Jami Aymacaña, M.B. Mora Paspuezan, D. Carrera Villacrés, O. Gutiérrez Cevallos, M. Masabanda Caisaguano, and V. Delgado Rodríguez. 2018. Caracterización físico-química del suelo del botadero de Portoviejo y análisis de la distribución espacial de cromo (VI), níquel, bromo y hierro. *FIGEMPA: Investigación y Desarrollo* 1(2): 10–19.
